# AIM: An Advanced Hybrid Inference Model Combining Clinical Rules and Lifelog-Based Learning for Health Risk Prediction

**DOI:** 10.3390/life16060928

**Published:** 2026-06-01

**Authors:** Junbeom Lee, Seyeon Kim, Nam-Hyeok Kim, Han-Gyeol Kim, Sinwoo Kim, Sungju Lee, Sungwook Yu, Jae-Min Park, Ji-Won Lee, Taikyeong Jeong

**Affiliations:** 1 School of Artificial Intelligence Convergence, Hallym University, Chuncheon 24252, Republic of Korea; 2 Department of Software Engineering, Sangmyung University, Cheonan 31066, Republic of Korea; 3 School of Electrical and Electronics Engineering, Chung-Ang University, Seoul 06974, Republic of Korea; 4 Department of Family Medicine, Uijeongbu Eulji Medical Center, Eulji University, Uijeongbu 11759, Republic of Korea; 5 Department of Family Medicine, Severance Hospital, Yonsei University College of Medicine, Seoul 03722, Republic of Korea; 6 Institute of Innovation in Digital Healthcare, Yonsei University, Seoul 03722, Republic of Korea; 7 Department of Psychiatry, College of Medicine, Hallym University, Chuncheon 24252, Republic of Korea

**Keywords:** symbolic reasoning, metabolic syndrome, risk assessment, long-term and short-term facts, lifelog data, random forest, rule-based expert system

## Abstract

**Background**: Early identification of metabolic health risk is important for preventive intervention, but routine laboratory testing is not always available in everyday health-management environments. Artificial intelligence models can estimate risk from accessible variables, but purely data-driven models may provide limited clinical interpretability. **Objective**: This study presents the Advanced Hybrid Inference Model (AIM), a clinically interpretable screening support framework that combines biomarker estimation, Random Forest-based risk prediction, and rule-based clinical interpretation. **Methods**: AIM was intentionally implemented as a three-stage, Random Forest-centered pipeline: (1) Selected anthropometric and demographic variables were used to estimate clinically relevant metabolic biomarkers when direct measurements were unavailable. (2) A Random Forest model generated metabolic risk estimates from measured or estimated biomarkers and clinical variables. (3) Rule-based interpretation mapped the model outputs and biomarker thresholds to clinically meaningful risk-support messages. **Results**: Experimental validation was conducted using clinically collected datasets under class-imbalanced conditions. The results indicate that the proposed framework showed exploratory potential for identifying high-risk patterns. These findings suggest that the AIM framework may be useful as a screening-oriented approach. **Conclusions**: AIM should be interpreted as an exploratory clinical screening support framework that prioritizes interpretability, structured rule-based reasoning, and risk prioritization rather than a diagnostic classifier or universally superior prediction model.

## 1. Introduction

According to the World Obesity Atlas (WOF), the population classified as obese, defined by a body mass index (BMI) of 30 or higher, has shown a steady increase. The latest data presented in the Atlas indicate that rapid lifestyle transitions and urbanization have significantly exacerbated the global obesity epidemic, with the affected population projected to reach approximately 1 billion by 2025 and to increase further by 2030 [1].

Within this context, metabolic syndrome has emerged as one of the most prevalent chronic conditions relative to the global population. It is estimated that approximately 20–25% of the world’s population is affected by metabolic syndrome [2]. Epidemiological investigations conducted between 1998 and 2007 reported a marked increase in prevalence, from 22.4% to 29.0% in men and from 27.9% to 32.9% in women, respectively [3]. This rapid growth suggests that metabolic syndrome is likely to impose a substantial socioeconomic burden in the near future.

Metabolic syndrome is widely recognized as a condition with high preventability when risk factors are identified at an early stage and appropriate interventions, such as lifestyle modification, are implemented before disease progression. However, accurate diagnosis of metabolic syndrome typically requires laboratory-based examinations, limiting continuous monitoring and management in everyday settings.

To address these challenges, recent research has increasingly focused on disease prediction and health management systems based on medical artificial intelligence. Nevertheless, many existing approaches rely predominantly on data-driven learning models, which often fail to adequately incorporate explicitly defined and clinically validated diagnostic rules. As a result, such models may suffer from limited interpretability and reduced clinical transparency. Moreover, approaches that treat disease risk assessment as a simple classification task are insufficient to capture the dynamic nature of individual health states and the complexity of comorbid disease environments.

In this study, we propose a hybrid artificial intelligence inference engine that structurally integrates clinically derived diagnostic criteria and expert knowledge into a rule-based artificial intelligence expert system and further combines this system with data-driven learning models utilizing lifelog and anthropometric data. The proposed framework distinguishes clinically stable diagnostic knowledge as long-term facts, while treating continuously collected lifelog data as short-term facts. This design enables the inference structure to reflect temporal changes in individual health conditions in a systematic manner.

Based on this concept, we introduce an advanced artificial intelligence inference engine termed the Advanced Hybrid Inference Model (*herein after called*, *AIM*). In this study, AIM is implemented as a structured three-stage inference pipeline consisting of (1) biomarker estimation from anthropometric variables, (2) Random-Forest-based metabolic risk prediction, and (3) rule-based clinical interpretation and recommendation generation. The proposed AIM is designed to evaluate disease risk in environments where multiple conditions—such as metabolic syndrome, dyslipidemia, and hypertension—interact simultaneously. By leveraging personal anthropometric information (e.g., height and weight) and personal healthcare records (PHRs), the system performs stepwise prediction of blood-based clinical indicators and subsequent disease risk assessment. This approach explicitly accounts for data accessibility constraints commonly encountered in real-world healthcare settings and provides a practical framework for disease risk evaluation.

Ultimately, the AIM-based inference engine is designed to translate disease risk assessment outcomes into personalized and interpretable health recommendation messages, supporting their potential utility for exploratory high-risk screening. Rather than aiming for overall performance improvement, this study focuses on improving sensitivity for high-risk case identification.

We hypothesize that explicitly separating long-term clinical knowledge from short-term lifelog data, and integrating them within a dual-driven inference architecture, will lead to positive outcomes. In particular, we expect the proposed model to achieve a higher F1-score in identifying high-risk cases compared to conventional rule-based or purely data-driven models.

The main contributions of this paper are summarized as follows:

- We present a simplified and clearly defined AIM framework centered on Random Forest-based metabolic risk estimation and rule-based interpretation.
- We formalize clinically defined disease assessment rules are structured as a rule-based expert system and systematically combined with machine learning and deep learning models, enabling the coexistence of knowledge-driven reasoning and statistical learning within a unified inference framework.
- We implement a staged screening support pipeline that uses accessible anthropometric and clinical variables to estimate metabolic risk when direct laboratory measurements may be incomplete.
- We provided a transparent process and utilized an proposed hybrid approach based on it to identify the practical limitations of data accessibility.
- We discuss the role of AIM as an exploratory screening support tool that explicitly distinguishes long-term facts and short-term facts for health risk assessment.

The outputs of the proposed inference engine are automatically converted into interpretable and personalized health recommendations, enhancing the practical usability and explainability of AI-based healthcare decision-support systems.

The remainder of this paper is organized as follows: Section 2 reviews related studies on medical artificial intelligence, rule-based expert systems, and hybrid inference approaches, highlighting their strengths and limitations in clinical risk assessment. Section 3 presents the structured representation of clinically validated disease assessment rules and describes the design of a rule-based expert system that models such knowledge as long-term facts. Section 4 formulates the proposed sequential dual drive-way learning process and provides a mathematical and algorithmic description of the Advanced Hybrid Inference Model (AIM), including its parallel inference architecture and residual-style fusion strategy. Section 5 describes the system-level implementation of the machine learning-based disease risk prediction model, detailing the integration of blood biomarker estimation, random forest-based risk classification, and expert-system-driven inference within the hybrid framework. Section 6 presents the experimental results and performance analysis, evaluating the effectiveness of the proposed model using clinical and lifelog datasets under class-imbalanced conditions. Finally, Section 7 concludes the paper by summarizing the main contributions and discussing the clinical significance, limitations, and future research directions of the proposed hybrid AI inference engine.

## 2. Related Work

In recent years, rapid advances in medical AI have fueled the development of diagnostic and clinical decision support systems. These systems analyze large-scale clinical data to predict disease risks and assist healthcare professionals in decision-making. A core component of this process is inference, which derives actionable insights by combining patient data with domain-specific knowledge [4,5].

Inference approaches in medical AI can be broadly categorized into rule-based inference and statistical inference. Rule-based inference, which is commonly employed in expert systems, relies on explicitly defined and clinically validated rules and medical knowledge to perform decision-making tasks [6,7,8,9,10,11]. Rule-based approaches offer high interpretability and consistency, yet they struggle to capture complex data patterns or time-varying conditions. Conversely, statistical inference—driven by machine learning and deep learning—excels at modeling complex nonlinear relationships from large-scale data. However, these data-driven methods often lack interpretability and face challenges in explicitly incorporating established clinical decision criteria.

To overcome these limitations, an increasing number of studies have explored hybrid artificial intelligence systems that combine rule-based and statistical inference mechanisms. One representative example is IBM Watson, a well-known medical AI platform. Watson for Oncology has been designed to analyze extensive clinical trial data and medical literature in order to provide personalized treatment recommendations based on individual patient information [12,13,14,15,16,17]. By integrating patient medical histories, genomic data, and imaging information, Watson aims to support clinical decision-making through large-scale data processing combined with knowledge-based inference. Despite its significance as a hybrid AI application, such systems are typically tailored to specific disease domains and exhibit structural limitations in reflecting continuously evolving health conditions in daily life.

Meanwhile, AI technologies have also been widely applied beyond disease diagnosis, particularly in drug discovery and life sciences. Recent studies have reported significant reductions in drug development timelines through AI-driven identification of drug candidate molecules [18]. In the field of protein structure prediction, DeepMind’s AlphaFold has been recognized as a landmark achievement [19]. By predicting protein structures from amino acid sequences, this approach has substantially advanced the understanding of molecular interactions in biological systems. The latest AlphaFold-3 model further demonstrates potential contributions to disease research and environmental problem-solving [20]. However, these studies primarily address molecular-level or domain-specific biological problems, with relatively less emphasis on individual-level disease risk assessment and lifestyle-based health management.

Early detection and prevention of disease are widely recognized as critical objectives in modern medicine, as they play a key role in slowing disease progression and improving treatment outcomes [21]. Accordingly, disease-specific databases and diagnostic systems have been developed and deployed in clinical settings. More recently, research efforts combining time-series data with machine learning algorithms have gained attention for early disease risk detection [22]. For example, Jeong et al. demonstrated the feasibility of early detection of mental and neurological disorders by integrating EEG and functional near-infrared spectroscopy (fNIRS) data using a hybrid framework incorporating shapelet analysis and dynamic time warping algorithms [8]. Such studies highlight the potential of hybrid approaches that integrate multiple data sources for disease risk analysis.

In addition to data-driven methodologies, theoretical frameworks have been proposed to explain disease onset and progression. Fleming’s theory [23] emphasizes the role of lifestyle factors, particularly dietary patterns, in the prevention and reversal of cardiovascular diseases. However, this theory is largely derived from analyses of Western populations, which limits its generalizability across diverse demographic groups. This limitation underscores the need for more flexible disease risk assessment models that can simultaneously account for individual lifestyle factors and clinically established diagnostic criteria.

The proposed differential dual-drive inference model is conceptually related to ensemble learning, residual networks, and multi-view learning; however, it differs fundamentally in both architectural intent and inference semantics.

Ensemble learning typically combines multiple independently trained models by averaging or voting their outputs to evaluate predictive architecture and robustness. While effective, ensemble methods treat each model as an independent estimator without explicitly modeling the relationships or differences among them. In contrast, the proposed dual-drive architecture is trained jointly and explicitly structured to capture differential information between parallel inference pathways. Rather than aggregating independent predictions, the auxiliary pathway is designed to learn residual or complementary representations relative to the dominant pathway, which are selectively fused through a residual-style formulation.

Residual networks (ResNets) introduce skip connections to facilitate gradient flow and enable deep architectures by learning residual mappings within a single network. Although the residual-style fusion used in this study is mathematically related, the conceptual role is different. Residual networks operate within a single inference stream, whereas the proposed model employs two structurally independent inference pathways with separate parameter sets. The residual formulation in the proposed architecture is used at the model-level rather than the layer-level, allowing explicit separation between dominant and auxiliary inference flows that correspond to distinct representational roles.

Multi-view learning seeks to exploit multiple feature views or modalities by learning from heterogeneous input spaces. In contrast, the proposed dual-drive model processes the same input space through parallel pathways to learn complementary representations. This design enables the model to capture heterogeneous interactions among clinical biomarkers without requiring additional data modalities. The regularization-based separation further distinguishes the proposed approach by encouraging divergence between learned representations while maintaining a shared prediction objective.

Overall, while sharing mathematical similarities with existing paradigms, the proposed differential dual-drive architecture introduces a novel combination of residual-style fusion and regularized pathway separation tailored for explainable and robust disease risk prediction.

Synthesizing the above related studies, prior research on medical artificial intelligence has largely relied on approaches biased toward either rule-based inference or data-driven learning. Consequently, there remains a relative lack of integrated inference architectures that simultaneously incorporate clinically validated diagnostic rules and time-varying lifelog data reflecting an individual’s evolving health conditions. This limitation becomes more pronounced in chronic disease settings, where multiple pathological factors and comorbidities interact; in such environments, a single inference paradigm is often insufficient to explain disease risk in a comprehensive and clinically meaningful manner.

To address this gap, this paper proposes a hybrid artificial intelligence inference engine, termed the Advanced Hybrid Inference Model (AIM), which combines a rule-based expert system with statistical learning models. The proposed approach models clinically stable diagnostic criteria as long-term facts, while integrating lifelog-based time-series signals as short-term facts, thereby enabling the inference process to capture both the consistency of clinical knowledge and the temporal dynamics of individual health states. This hybrid inference structure is designed to mitigate the interpretability limitations of purely data-driven models while enabling integrated inference between rule-based knowledge and data-driven learning, providing a structured framework for disease risk assessment with particular emphasis on high-risk case identification.

In the next section, we first organize and formalize the disease classification criteria used in clinical decision-making, and then present the detailed architecture of the rule-based expert system for lifelog data processing and the proposed AIM framework.

As summarized in Table 1, rule-based inference provides high interpretability but lacks adaptability to temporal health changes, while data-driven approaches excel at pattern learning but suffer from limited clinical interpretability. The proposed hybrid inference paradigm combines the complementary strengths of both approaches, enabling clinically consistent, temporally adaptive, and interpretable health risk assessment.

## 3. Clinical Knowledge Representation and Expert System Design

In this section, we address the limitations of existing medical artificial intelligence inference paradigms discussed in Section 2 and summarized in Table 1 by presenting the design of a rule-based expert system as a core component of the proposed hybrid inference framework. While rule-based approaches ensure interpretability and clinical consistency and data-driven models provide strong pattern learning capabilities, neither paradigm alone is sufficient to support personalized health risk assessment under dynamically changing individual conditions.

Accordingly, clinically validated disease assessment criteria are systematically structured as explicit rules and modeled as long-term facts, forming the foundational knowledge layer of the Advanced Hybrid Inference Model (AIM). This rule-based expert system enables consistent and explainable reasoning grounded in established medical knowledge and serves as a stable interface for subsequent integration with lifelog-derived short-term facts in later stages of the hybrid inference process.

### 3.1. Clinical Rule Representation for Disease Risk Assessment

This subsection focuses on the systematic representation of clinically defined disease assessment criteria as explicit rules within a rule-based expert system. The primary objective of this step is not to perform clinical diagnosis but to formalize established clinical knowledge into a structured and interpretable form that can be utilized as long-term facts in the proposed hybrid inference framework.

In medical artificial intelligence systems, clinical assessment criteria are typically defined based on long-standing consensus and validated guidelines. Such criteria provide stable and interpretable decision boundaries, making them suitable for representation as rule-based knowledge. In the proposed hybrid inference model (AIM), these clinically defined criteria are modeled as long-term facts, which serve as the foundational knowledge layer for subsequent inference processes.

#### 3.1.1. Definition of Long-Term Facts in Disease Risk Assessment

In this study, long-term facts refer to objective and stable disease assessment criteria that are derived from established clinical guidelines and expert consensus. Unlike dynamically changing lifestyle or behavioral data, long-term facts represent invariant reference knowledge used to assess disease risk. These facts are not learned from data but are explicitly defined and encoded as rules within an expert system.

Accordingly, disease risk assessment in the proposed framework is conducted by applying rule-based reasoning over long-term facts, ensuring consistency and interpretability. This approach aligns with conventional expert system methodologies while enabling seamless integration with data-driven components in later stages.

#### 3.1.2. Case Study: Rule Representation for Metabolic Syndrome

To illustrate the representation of clinical rules as long-term facts, metabolic syndrome is selected as a representative case. Metabolic syndrome is a chronic condition characterized by the coexistence of multiple risk factors and is commonly assessed using a set of well-defined clinical criteria.

Table 2 summarizes the clinically defined assessment criteria for metabolic syndrome, which are represented as explicit rules in the proposed expert system [5,6,7,8,9,10].

According to the criteria shown in Table 2, metabolic syndrome is identified when three or more of the five conditions are satisfied. In this study, the number of satisfied conditions is denoted as n, and the risk level is categorized as follows: *n* = 0 indicates normal, *n* = 1 indicates caution, *n* = 2 indicates risk, and *n* ≥ 3 indicates metabolic syndrome. These categorizations are encoded as rule-based conditions and stored as long-term facts within the expert system.

It should be noted that this classification process does not constitute a clinical diagnosis but rather serves as a structured rule-based risk assessment mechanism designed for integration into the AI inference engine. The classification is performed using either predicted or measured biomarker values depending on the operational mode of the framework.

#### 3.1.3. Extension to Multiple Diseases: Dyslipidemia and Diabetes

To support multi-disease risk assessment, the proposed framework extends the rule representation beyond metabolic syndrome to include dyslipidemia and diabetes, which frequently co-occur with metabolic syndrome.

Table 3 presents the clinically defined assessment criteria for dyslipidemia [11], organized into multiple risk levels. These criteria are similarly encoded as explicit rules and treated as long-term facts in the expert system.

Table 3 summarizes dyslipidemia risk levels based on interval-defined lipid thresholds derived from NCEP ATP III guidelines [12,13,14,15,16]. Each lipid parameter is categorized into mutually exclusive risk strata without logical disjunctions, enabling direct integration into rule-based inference models.

In addition, diabetes-related long-term facts are defined based on clinically accepted criteria, as summarized in Table 4.

Table 4 presents interval-based glycemic thresholds for HbA1c, fasting plasma glucose, and the 2 h oral glucose tolerance test, as defined by ADA and WHO guidelines [17,18,19,20]. These thresholds are operationalized in Table 4 as three discrete diabetes risk stages—normal, pre-diabetes, and diabetes—and are implemented as rule-based conditions within the long-term knowledge layer of the proposed inference framework.

#### 3.1.4. Summary and Role of Clinical Rules in the AIM Framework

Through the representation of metabolic syndrome, dyslipidemia, and diabetes assessment criteria, this subsection demonstrates how clinically defined knowledge can be systematically structured as long-term facts within a rule-based expert system. The presented disease criteria follow conventional clinical assessment guidelines while being reformulated into a machine-interpretable rule structure.

This rule representation process constitutes a preparatory step for extending disease assessment into the artificial intelligence inference domain. By modeling multiple disease criteria as long-term facts, the proposed framework establishes a consistent and interpretable knowledge foundation that supports subsequent integration with dynamically changing short-term facts derived from lifelog and sensor data.

### 3.2. HDL Normalization and Rule-Based Dyslipidemia Inference

To integrate clinical high-density lipoprotein (HDL) cholesterol measurements with the proposed rule-based inference framework, HDL values are normalized to a continuous range of [0, 1] using a min–max normalization scheme grounded in clinically meaningful thresholds. This normalization enables consistent integration of heterogeneous data modalities, including clinical biomarkers and lifelog-derived lifestyle indicators, within a unified inference model.

The HDL normalization formula is defined as follows:

(1) $$HDL_{\mathrm{norm}}=\frac{HDL_{\mathrm{raw}}-HDL_{min}}{HDL_{max}-HDL_{min}}$$

where HDL_raw_ denotes the measured HDL cholesterol value in mg/dL, HDL_min_ represents the lower bound of the clinically relevant HDL range, and HDL_max_ denotes the upper bound of the same range.

Based on established clinical guidelines from the National Cholesterol Education Program Adult Treatment Panel III (NCEP ATP III) [6,7,8] and the American Diabetes Association (ADA) [9], the normalization bounds are defined as

(2) $$HDL_{min}\;=\;40\;\;\mathrm{mg}/\mathrm{dL},\;{HDL}_{max}\;=\;80\;\;\mathrm{mg}/\mathrm{dL}$$

Accordingly, the implemented normalization equation used in the proposed system becomes

(3) $$HDL_{\mathrm{norm}}=\frac{HDL_{\mathrm{raw}}-40}{40}$$

To ensure robustness against outliers and extreme clinical values, the normalized HDL value is constrained to the interval [0, 1] through saturation handling:

(4) $$HDL_{norm}=min\left(1,max\left(0,{HDL}_{norm}\right)\right)$$

The resulting normalized HDL value is subsequently mapped to interval-defined dyslipidemia inference rules. These rules categorize dyslipidemia status into three discrete states—At risk, Moderate, and Optimal—based on clinically interpretable HDL ranges:

- $HDL_{norm}$ ≤ 0.2: Dyslipidemia **At risk**;
- 0.2 < $HDL_{norm}$ < 0.5: Dyslipidemia **Moderate**;
- $HDL_{norm}$ ≥ 0.7: Dyslipidemia **Optimal**.

The quantitative correspondence between normalized HDL values, raw clinical measurements, and rule-based inference outcomes is summarized in Table 5.

Through this normalization and rule-encoding process, HDL cholesterol values are transformed into interpretable symbolic representations that can be directly incorporated into the long-term fact base of the proposed system. The normalized HDL values serve as triggers for interval-defined dyslipidemia rules, enabling consistent, transparent, and explainable reasoning across heterogeneous data modalities, including clinical laboratory results and lifelog-derived behavioral indicators.

## 4. Conceptual Extension of Hybrid Inference Framework

The dual-drive formulation presented in this section serves as a conceptual extension of the hybrid inference framework, while the primary experimental validation focuses on the hybrid Random Forest (RF)-based pipeline.

The proposed pipeline categorizes variables into observed inputs, predicted intermediate biomarkers (e.g., LDL, HDL, TG, and TC), and final disease risk outputs. When laboratory measurements are available, biomarkers are directly incorporated as observed inputs rather than predicted intermediates.

### 4.1. Algorithm

The proposed inference framework models lipid biomarker prediction as a sequential and bidirectional estimation process, in which previously predicted values are explicitly reused as inputs for subsequent models. When clinical biomarkers are available, these are directly used as observed inputs. Let ***X*** denote the anthropometric and demographic input vector:

(5) X=[AGE,SEX,BMI,PSQI,Muscle,Fat,SBP,DBP,HR,Waist,Fat%,WHR]

**Step 1: Initial LDL Prediction (Model 1 Drive-Way)**

The first model in the Model 1 drive-way predicts LDL cholesterol solely based on the input vector X:

(6) $$LDL_{PRE}^{\left(1\right)}=f_{LDL}\left(X;\;\theta_{LDL}\right)$$

where $f_{LDL}$(⋅) denotes a learnable prediction function trained using ground-truth LDL values. This step establishes the initial dominant lipid estimate, which is treated as prior knowledge in subsequent stages.

**Step 2: HDL Prediction with Feedback (Model 2 Drive-Way)**

The predicted LDL value is then fed back and combined with the original input to predict HDL cholesterol through the Model 2 drive-way:

(7) $$HDL_{PRE}^{\left(1\right)}=f_{HDL}\left(X,LDL_{PRE}^{\left(1\right)};{\;\theta }_{HDL}\right)$$

This formulation explicitly models the dependency of HDL on previously predicted LDL values. The bidirectional arrows in the architecture indicate that prediction (pre) and parameter update (training) occur iteratively.

**Step 3: Triglyceride Prediction Using Cascaded Estimates**

Next, both predicted LDL and HDL values are reused to estimate triglyceride levels:

(8) $$TG_{PRE}^{\left(1\right)}=f_{TG}\left(X,LDL_{PRE}^{\left(1\right)},HDL_{PRE}^{\left(1\right)};{\;\theta }_{TG}\right)$$

At this stage, the model captures higher-order interactions among lipid components by incorporating multiple prior predictions as structured inputs.

**Step 4: Total Cholesterol Prediction via Progressive Aggregation**

The prediction process is further extended to total cholesterol by aggregating all previously estimated lipid biomarkers:

(9) $${TC}_{PRE}^{\left(1\right)}=f_{TC}\left(X,LDL_{PRE}^{\left(1\right)},{HDL}_{PRE}^{\left(1\right)},TG_{PRE}^{\left(1\right)};{\;\theta }_{TC}\right)$$

This progressive aggregation enables the model to learn clinically meaningful dependencies among lipid variables rather than treating them as independent targets.

**Step 5: Final LDL Refinement through Bidirectional Update**

Finally, the model performs a refinement step in which LDL cholesterol is re-estimated using the full set of predicted lipid values:

(10) $$LDL_{PRE}^{LAST}=f_{LDL}^{*}\left(X,HDL_{PRE}^{\left(1\right)},TG_{PRE}^{\left(1\right)},TC_{PRE}^{\left(1\right)};{\;\theta }_{LDL}^{*}\right)$$

This step produces a converged LDL estimate that reflects bidirectional information flow across the entire lipid prediction chain.

To avoid ambiguity, the proposed framework explicitly distinguishes two operational modes: (i) measurement-free mode, where biomarkers are treated as predicted intermediate variables, and (ii) measurement-assisted mode, where biomarkers are treated as observed inputs.

### 4.2. Interpretation of the Sequential Formulation

Through this formulation, each lipid biomarker prediction is expressed as a conditional function of both the original anthropometric inputs and previously predicted biomarkers:

(11) $$y_{k}\;=\;f_{k}\left(X,\;y_{1},\;\ldots ,\;y_{k-1}\right)$$

This structure transforms lipid estimation into a structured inference problem rather than a set of independent regression tasks. The bidirectional interaction between prediction and training ensures that earlier estimates influence later predictions while being iteratively corrected through learning.

Algorithm 1 summarizes the complete training and inference procedure of the proposed Sequential Dual Drive-Way learning process. Unlike independent regression models, each lipid biomarker prediction is recursively conditioned on previously estimated values and refined through a bidirectional update mechanism. The multi-output loss function enables joint optimization across all lipid variables, allowing the model to capture clinically meaningful interdependencies rather than treating biomarkers as independent targets.
**Algorithm 1.** Pseudo-code of Sequential Dual Drive-Way Lipid Estimation Framework**Input:**Anthropometric and demographic vector X Ground-truth lipid biomarkers Y = {LDL, HDL, TG, TC}**Output:**Refined lipid estimates {$LDL_{PRE}^{LAST},$
$HDL_{PRE}^{\left(1\right)},$
$TG_{PRE}^{\left(1\right)},$
$TC_{PRE}^{\left(1\right)}$}01:Initialize model parameters $\theta_{LDL},$${\;\theta }_{HDL},$${\;\theta }_{TG},$${\;\theta }_{TC},$${\;\theta }_{LDL}^{*}$
02:Set learning rate *η* and convergence criterion *ε*03:**for** each training epoch **do**04:     $LDL_{PRE}^{\left(1\right)}$ ← $f_{LDL}\;$(X; $\theta_{LDL}$)05:        $HDL_{PRE}^{\left(1\right)}$ ← $f_{HDL}$(X, ${LDL}_{PRE};$${\;\theta }_{HDL}$)06:        $TG_{PRE}^{\left(1\right)}$ ← $f_{TG}$(X, $LDL_{PRE}^{\left(1\right)},$
$HDL_{PRE}^{\left(1\right)};$${\;\theta }_{TG}$)07:        $TC_{PRE}^{\left(1\right)}$ ← $f_{TC}$(X, $LDL_{PRE}^{\left(1\right)},$$\;HDL_{PRE}^{\left(1\right)},$
$TG_{PRE}^{\left(1\right)};$${\;\theta }_{TC}$)08:     $LDL_{PRE}^{LAST}$ ← $f_{LDL}*\;$(X, $HDL_{PRE}^{\left(1\right)},$
$TG_{PRE}^{\left(1\right)},$
$TC_{PRE}^{\left(1\right)}$}; ${\;\theta }_{LDL}^{*}$)09:        L = Σ_k_ ||Y_k_ − Y_k PRE_||^2^, where k ∈ {${LDL}_{LAST},$ HDL, TG, TC}10:θ**←** θ** − *η* ∇**θ**L**11:**if** |ΔL| < ε **then**; break12:**end for**13:**Return** final predicted lipid biomarkers

In particular, the proposed framework integrates sequential forward propagation with bidirectional refinement to effectively model interdependencies among lipid biomarkers. Unlike conventional cascade or stacking approaches, where intermediate predictions are passively reused, the proposed architecture introduces an explicit refinement stage that re-estimates dominant biomarkers using aggregated intermediate outputs. This design mitigates error accumulation and reduces forward propagation bias.

Furthermore, by jointly optimizing all lipid targets within a unified learning framework, the proposed approach improves gradient consistency and enhances convergence stability compared to independently trained regression models. 

While Section 4 presents the mathematical and algorithmic formulation of the proposed sequential dual drive-way learning process, Section 5 focuses on its system-level implementation and the overall architectural design of the hybrid AI inference engine, which integrates machine learning with rule-based expert reasoning.

## 5. Machine Learning-Based Risk Prediction Model Implementation

While the proposed model focuses on data-driven prediction of blood biomarkers and metabolic syndrome risk, practical clinical judgment involves additional layers of reasoning, including rule-based interpretation aligned with clinical standards, the incorporation of lifestyle factors, and longitudinal decision-making informed by previously accumulated results.

### 5.1. Inference Architecture and Design Criteria

This section describes the machine learning-based disease risk prediction model responsible for data-driven risk estimation within the proposed inference architecture. The dual-drive formulation supports the conceptual framework, while the Random Forest model serves as the primary predictive component in the practical implementation of the proposed system. The machine learning component in this study is not intended to replace clinically established rule-based decision-making; rather, it is designed to generate quantitative risk indicators that serve as inputs for subsequent expert system inference. Accordingly, this section focuses on the construction of input variables, including the prediction of blood-based clinical measurements, the selection of learning models, and the overall process for metabolic syndrome risk estimation.

#### 5.1.1. Input Feature Construction

The input variables for the machine learning-based risk prediction model are organized into two main categories. The first category consists of predicted blood-based clinical measurements obtained from the multivariate linear regression models described in Section 3.1, including triglycerides, high-density lipoprotein (HDL) cholesterol, and fasting glucose. The second category includes clinically relevant anthropometric and demographic variables that are widely recognized as important indicators for metabolic syndrome risk assessment, namely waist circumference, blood pressure, sex, and age.

This variable configuration is designed to reflect key indicators commonly used in actual clinical diagnostic workflows while simultaneously accounting for data availability and prediction stability. In particular, the predicted blood-based measurements serve as intermediate inference outcomes derived from anthropometric data, enabling the machine learning model to utilize clinically meaningful summarized information rather than relying solely on raw input variables. This design choice facilitates a more structured and interpretable risk prediction process that aligns with established clinical reasoning.

#### 5.1.2. Risk Prediction Model Selection

To predict metabolic syndrome risk, this study employs the Random Forest algorithm. Random Forest constructs an ensemble of multiple decision trees, thereby mitigating overfitting and effectively learning nonlinear interactions among input variables. It is particularly well suited for real-world clinical datasets and provides screening results due to its tree-based structure [4]. These characteristics make it appropriate for metabolic syndrome risk assessment, where diverse clinical variables interact in a complex and interdependent manner.

In addition, Random Forest provides quantitative estimates of variable importance, offering a degree of interpretability for the prediction results. This property is consistent with the explainable inference framework pursued in this study and facilitates seamless integration with the subsequent rule-based expert system, where learned risk patterns can be linked to clinically interpretable decision rules.

#### 5.1.3. Model Training and Output Representation

The Random Forest model is trained using the learning dataset to predict metabolic syndrome risk in the form of multiple classes or ordinal risk levels. Rather than producing a single binary classification outcome, the model outputs quantitative scores or probability values that represent the estimated risk level. These outputs are subsequently interpreted in combination with clinically defined criteria during the rule-based inference stage and are not used as direct final diagnostic results.

Considering that the outputs of the machine learning model serve as inputs to the expert system, this study prioritizes the stability and consistency of the predicted risk indicators. This design ensures that data-driven predictions do not conflict with rule-based clinical judgments but instead function in a complementary manner, enabling coherent integration between statistical learning and expert system inference.

#### 5.1.4. Role of Machine Learning in the Hybrid Inference Framework

In the proposed inference framework, the machine learning-based risk prediction model plays a supportive yet essential role. Specifically, the machine learning component learns latent risk patterns from large-scale data and provides quantitative risk indicators, while the expert system integrates these indicators with clinically established rules and long-term knowledge to perform the final decision-making process.

**Figure 1:**
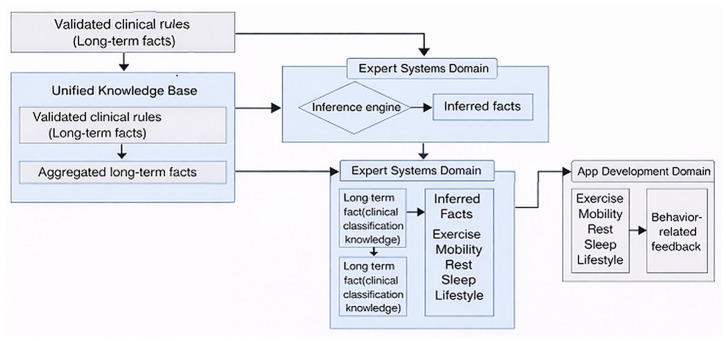
Architecture of a rule-based expert system integrating validated clinical rules and long-term facts to generate inferred knowledge and behavior-oriented feedback for app-level lifestyle interventions.

Figure 1 illustrates the overall architecture of the artificial intelligence expert system proposed in this study. Clinically validated diagnostic rules are stored in an integrated knowledge base as long-term facts, while blood-based clinical measurements predicted by the machine learning models, along with anthropometric information, are used as inputs to the inference engine.

Within the expert system layer, rule-based inference is performed based on the combination of long-term facts and newly acquired input data, resulting in the generation of inferred facts. These inferred results are subsequently accumulated as part of the long-term knowledge base, thereby maintaining continuity and consistency in the system’s decision-making process. The final inference outcomes are then translated into personalized behavioral recommendations—such as guidance on exercise, physical activity, rest, sleep, and lifestyle habits—which are utilized within the application service layer.

Through this architecture, the proposed disease prediction framework extends beyond single-time-point risk estimation and realizes an explainable and scalable disease risk assessment and recommendation system that continuously incorporates both clinical knowledge and individual lifestyle patterns.

### 5.2. Machine Learning-Based Risk Prediction Model

Based on the hybrid advanced inference engine described above, the proposed artificial intelligence-based disease prediction model is structured into two main stages. The first stage focuses on predicting blood-based clinical measurements from anthropometric data. The second stage utilizes the predicted blood-based measurements in conjunction with clinical information to assess metabolic syndrome risk and to generate personalized exercise recommendation messages.

#### 5.2.1. Inferencing Pipeline

In the first stage, correlations between the anthropometric data and blood-based clinical measurements included in the dataset were analyzed. The anthropometric variables comprised body mass index (BMI), waist circumference, height, weight, body fat mass, blood pressure, body fat percentage, and muscle mass, while the blood-based measurements included triglycerides, high-density lipoprotein (HDL) cholesterol, and fasting glucose. Among these variables, waist circumference, BMI, height, weight, and body fat mass—showing relatively strong correlations with blood-based measurements—were selected as the primary predictive variables. To model the relationships between anthropometric and blood-based measurements, a multivariate linear regression model was employed, enabling the prediction of triglycerides, HDL cholesterol, and fasting glucose from anthropometric data.

The adoption of a multivariate linear regression model in this stage was motivated by the need to ensure not only predictive architecture but also clinical interpretability. By explicitly representing the relationships between anthropometric variables and blood-based measurements in the form of mathematical equations, the predicted outcomes can be utilized as clinically explainable intermediate inference results rather than opaque black-box outputs. These first-stage prediction results subsequently serve as key input variables for the metabolic syndrome risk assessment performed in the next stage.

In the second stage, the blood-based measurements predicted in the first stage (triglycerides, HDL cholesterol, and fasting glucose) are integrated with clinically important risk factors, including waist circumference, blood pressure, sex, and age, to estimate an individual’s metabolic syndrome risk. For this purpose, the Random Forest algorithm was employed due to its ability to effectively model nonlinear interactions among variables and to handle complex decision boundaries. Given that metabolic syndrome risk arises from the interaction of multiple clinical factors, Random Forest was considered an appropriate model for learning such multifactorial risk patterns in a stable manner.

However, the prediction results generated by the Random Forest model are not directly used as final diagnostic outcomes. Instead, this study introduces an expert system-based advanced inference engine to interpret data-driven predictions in conjunction with clinically validated rules and lifestyle-related information. In this framework, the machine learning model provides quantitative estimates of disease risk, while the final decision-making process is conducted through an inference structure that leverages established clinical rules and accumulated knowledge. This design enables interpretable disease risk assessment that reflects clinical context, rather than relying solely on categorical classification results.

In the final stage, the predicted disease risk and individual lifelog information are jointly considered to generate personalized exercise recommendation messages. Physical activity levels derived from lifelog data are evaluated based on moderate-to-vigorous aerobic activity, with a threshold of at least 150 min of physical activity per week [3]. This criterion reflects recommendations provided by the World Health Organization (WHO) and related clinical guidelines.

The lifelog-based physical activity criteria serve as a design constraint to clearly distinguish disease risk from lifestyle factors and to prevent excessive or clinically inappropriate exercise recommendations during message generation. Through this mechanism, the proposed recommendation system provides differentiated exercise guidance according to disease risk while supporting potential integration into daily health management.

**Figure 2:**
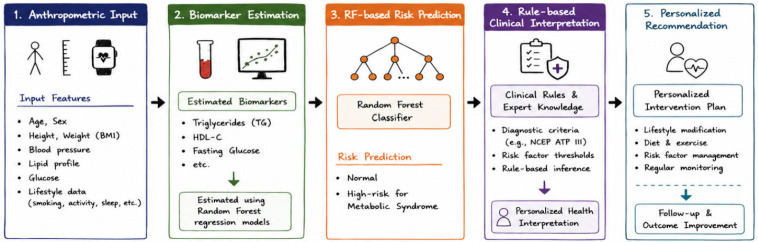
Simplified overview of the proposed AIM screening framework. Anthropometric and lifelog-derived inputs are first used for biomarker estimation, followed by Random Forest-based risk prediction and rule-based clinical interpretation.

Overall, the proposed disease prediction and recommendation model is designed as a staged inference pipeline consisting of prediction, assessment, and recommendation phases. This structure reflects the reality of clinical practice, where disease risk evaluation is not based on single-point numerical predictions but rather on the sequential interpretation of multiple clinical indicators and lifestyle-related information. Based on this staged inference design, the present study introduces an artificial intelligence expert system that integrates data-driven predictions with clinical rules, and the overall inference architecture is illustrated in the following Figure 2.

#### 5.2.2. Sequential Dual Drive-Way Architecture

Through this hybrid architecture, the proposed framework mitigates the limited explainability commonly associated with purely data-driven models while simultaneously alleviating the rigidity inherent in rule-based systems. As a result, the machine learning-based risk prediction model functions as a core component of the advanced inference engine, enabling disease risk assessment that satisfies both clinical validity and interpretability.

The motivation for adopting a dual-drive architecture arises from both clinical reasoning and machine learning considerations.

From a clinical perspective, disease risk assessment is rarely based on a single homogeneous reasoning process. Instead, clinicians often establish a primary risk judgment based on dominant biomarkers and subsequently refine this judgment by considering secondary or contextual factors. The proposed dual-drive model mirrors this process by structurally separating dominant and auxiliary inference flows and integrating them through a controlled fusion mechanism.

From a modeling standpoint, single-path deep networks are prone to overfitting dominant correlations in clinical data, potentially neglecting less frequent but clinically meaningful patterns. By introducing an auxiliary pathway constrained through regularization-based separation, the proposed model explicitly allocates representational capacity for capturing residual or heterogeneous interactions among biomarkers.

Moreover, unlike conventional ensemble approaches, the dual-drive architecture enables joint training and explicit interaction modeling between pathways, improving robustness without sacrificing interpretability. The residual-style fusion ensures that auxiliary information refines rather than overrides the primary inference, thereby maintaining stability and clinical plausibility of predictions. The outputs of the two pathways are combined through a residual-style fusion mechanism.

The final prediction is defined as

(12) $$y=f_{1}\left(x\right)+\lambda \;f_{2}\left(x\right)\;$$

where $f_{1}\left(x\right)$ represents the primary pathway and $f_{2}\left(x\right)$ denotes the auxiliary pathway, and λ controls the contribution of the auxiliary correction term. To further encourage differentiation between the two pathways during joint optimization, we introduce a regularized loss function defined as:

(13) $${L\;=\;L}_{pred}+\beta {\left\|\omega_{1}\;-\;\omega_{2}\right\|}^{2}$$

where $L_{pred}$ denotes the prediction loss, $\omega_{1}$ and $\omega_{2}$ represent the parameters of the two pathways, and β controls the strength of the regularization term. This regularization discourages trivial convergence of the two pathways toward similar parameter configurations. Figure 2 presents a differential dual-drive disease prediction model in which dominant and auxiliary inference pathways are explicitly separated, regularized, and fused through a residual-style formulation to generate a robust and interpretable disease risk estimate.

In summary, the dual-drive architecture is not an arbitrary extension but a principled design choice that aligns with clinical decision-making processes, enhances representational diversity, and supports robust, explainable disease risk prediction within a unified inference framework.

**Figure 3:**
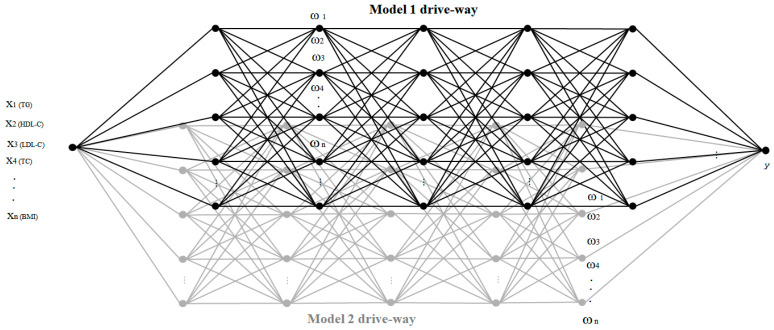
Overall architecture of the proposed disease prediction model with a differential dual-drive inference structure. Clinical biomarkers and anthropometric inputs are processed through two parallel pathways (Model 1 and Model 2 drive-ways), whose representations are fused to produce the final risk prediction y.

Figure 3 illustrates the overall architecture of the proposed AI-based disease prediction model, which is designed as a differential dual-drive inference structure within the Advanced Hybrid Inference Model (AIM). Let the clinical and anthropometric input vector be defined as

(14) $$x=\left[x_{1},x_{2},\ldots ,x_{n}\right],$$

where $x_{i}$ denotes individual biomarkers and anthropometric variables such as triglycerides (TG), HDL-C, LDL-C, total cholesterol (TC), and body mass index (BMI). These heterogeneous clinical inputs are simultaneously fed into two parallel inference pathways, referred to as Model 1 drive-way and Model 2 drive-way, which share the same input space but are parameterized independently.

Each pathway learns a nonlinear mapping from the input space to a latent representation:

(15) $$h_{1}=f_{1}\left(x;{\;\omega }_{1}\right),\;h_{2}=f_{2}\left(x;{\;\omega }_{2}\right),$$

where $f_{1}$ (⋅) and $f_{2}$ (⋅) denote deep neural network functions composed of multiple fully connected layers, and $\omega_{1}$ and $\omega_{2}$ represent the corresponding sets of learnable parameters. In Figure 2, the upper pathway is depicted using darker nodes and edges to indicate the dominant inference flow, while the lower pathway is illustrated using lighter nodes and edges to represent an auxiliary inference flow that captures complementary or residual patterns.

## 6. Experimental Results and Analysis

In this section, we present and analyze the experimental results to validate the proposed sequential and parallel deep learning-based inference framework for disease risk prediction. The effectiveness of the multi-stage LDL prediction process is evaluated using clinical and lifelog datasets.

### 6.1. Experimental Setup and Data Description

The dataset used in this study was retrospectively collected from patients who visited Yonsei University Severance Hospital based on electronic medical records (EMR) and associated clinical databases under Institutional Review Board (IRB) approval [4]. In addition, the data collection process involved routine clinical assessments conducted during hospital visits, reflecting real-world clinical practice for *five* years. The dataset consists of duplicated measurements obtained from individuals wearing wearable devices, with measurements conducted twice at *eight*-week intervals.

Initially, the dataset incorporates population-based cohort data, including the Korean Genome and Epidemiology Study (KoGES), which enhances the representativeness and generalizability of the proposed framework. Over the approximately *five* years of collection, all final datasets complied with relevant guidelines and regulations, and included clinical evaluations where hospital clinical activities were performed, with a final total of 695 samples retained. The final data used in this study consist of clinical and lifelog-based measurement data collected through repeated measurements. The use of the KOGES cohort, a well-established population-based epidemiological dataset, ensures that the model is trained and validated on heterogeneous, real-world data distributions. This contributes to improved robustness against sampling bias and supports the generalizability of the proposed framework beyond institution-specific or narrowly curated datasets as shown in Table 6. This repeated-measurement design provides a longitudinal structure that enables the assessment of temporal changes in lifelog-based health conditions.

The risk levels for metabolic syndrome prediction were formulated as a multi-class classification problem using one-hot encoding. The entire dataset (*n* = 695) was divided into a training set (486 samples) and a test set (209 samples) at the participant level to prevent data leakage. Training and test splits were performed at the participant level to prevent repeated measurements from appearing simultaneously in both sets. The reported 695 samples correspond to participant-level observations after preprocessing and exclusion. Also, we verified that repeated measurements collected at multiple visits were treated as independent temporally separated observations.

For metabolic syndrome classification, a binary evaluation subset was constructed. To ensure clinically meaningful group separation, only subjects with clearly defined Normal status group (*n* = 137, 0 conditions) and Metabolic Syndrome group (named MetS, *n* = 52, ≥3 conditions) were included, while intermediate-risk cases (1–2 conditions) were excluded.

As a result, the binary evaluation sub-dataset consisted of 189 participants, including 137 Normal group and 52 MetS group cases. All classification performance metrics were computed based on this sub-dataset. Performance metrics, including the confusion matrix, were computed on this filtered sub-dataset rather than the full test set of 209 samples. Training and test splits were consistently performed at the participant level across all experimental settings.

The baseline clinical and biochemical characteristics of the study population are presented in Table 7. The participants were categorized into two groups based on the number of satisfied metabolic conditions: the Normal group (0 condition) and the Metabolic Syndrome group (*n* = 52, ≥3 conditions).

Significant demographic and anthropometric differences were observed between the two groups. The MetS group was significantly older (54.7 ± 10.5 years) than the Normal group (39.4 ± 13.5 years, *p* < 0.001). Body Mass Index (BMI) and Waist Circumference (WC) were also markedly higher in the MetS group (27.4 ± 6.2 kg/m^2^ and 81.0 ± 11.9 cm, respectively) compared to the Normal group (*p* < 0.001). Although the proportion of male participants was higher in the MetS group (27.8%) than in the Normal group (21.2%), this difference did not reach statistical significance (*p* = 0.432).

Regarding clinical parameters, the MetS group exhibited significantly higher blood pressure and fasting glucose levels. Both systolic and diastolic blood pressure were significantly elevated in the MetS group (*p* < 0.001). Similarly, fasting glucose levels were substantially higher in the MetS group (112.2 ± 17.2 mg/dL) compared to the Normal group (91.3 ± 5.4 mg/dL, *p* < 0.001).

In the lipid profile analysis, TG were more than twice as high in the MetS group (194.9 ± 120.8 mg/dL) as in the Normal group (74.6 ± 24.4 mg/dL, *p* < 0.001), while HDL-C was significantly lower in the MetS group (*p* < 0.001). Notably, no significant differences were observed in LDL-C (*p* = 0.059) and TC (*p* = 0.138) between the two groups, suggesting that the metabolic risk in this study population is primarily driven by insulin resistance-related markers (TG, HDL-C, and glucose) rather than LDL-C levels. This sub-dataset was used to ensure a clinically meaningful binary comparison between clearly defined normal and metabolic syndrome groups.

All experiments were conducted on a workstation equipped with an Intel Core i7 processor, 32 GB RAM, and an NVIDIA RTX 3090 GPU. The models were implemented in Python 3.1 using PyTorch 2.3.1 and Scikit-learn libraries 1.5.1, and experiments were run on a Linux-based operating system.

### 6.2. Overall Performance Evaluation

To evaluate the effectiveness of the proposed hybrid inference framework, a Random Forest-based classification model was applied to a clinical dataset characterized by imbalanced class distributions. In the test set, the proportion of high-risk (positive) cases was relatively low (approximately 15–25%), while the remaining classes constituted the majority. Under this imbalance setting, the classification objective focused on potential utility for exploratory high-risk screening rather than maximizing overall four-class accuracy.

Therefore, conventional accuracy alone is insufficient to evaluate the clinical utility of the proposed screening framework under class-imbalanced conditions.

During training of the Random Forest algorithm, a grid search strategy was employed to determine optimal hyper-parameters. The number of trees was set to 16, the maximum tree depth to 8, and the minimum number of samples required for node splitting and leaf nodes to 8. After training, performance evaluation was conducted using the proposed artificial intelligence inference engine (AIM). In the first stage of the disease prediction process proposed in this paper, the dataset consisting of 695 patient samples was used to learn the relationships between anthropometric variables and blood-based clinical measurements defined in Section 3.2. Based on this training process, explicit relationship equations for each blood-based measurement were derived. The resulting regression equations for the predicted blood-based indicators are presented as follows, as components of the overall inference framework.

(16) $$Y_{TG}=109.79856115+\left(-93.92961098\right)x_{BMI}+13.87407286x_{Waist}+\left(-77.31087993\right)x_{Height}+169.01019209x_{Weight}+\left(-14.97816429\right)x_{Fat}$$

(17) $$Y_{HDL}=59.28345324+6.23389407x_{BMI}+\left(-5.74408617\right)x_{Waist}+5.1362362x_{Height}+\left(-11.78239958\right)x_{Weight}+2.60238202x_{Fat}$$

(18) $$Y_{Fasting\;Glucose}=99.48633094+\left(-3.9127618\right)x_{BMI}+3.41710074x_{Waist}+\left(-5.09336344\right)x_{Height}+9.35024781x_{Weight}+\left(-1.03578909\right)x_{Fat}$$

Equation (16) represents a multivariate linear regression model that estimates triglyceride (TG) levels ($Y_{TG}$) using body mass index (BMI), waist circumference, height, weight, and body fat percentage as independent variables. According to this equation, waist circumference and weight exhibit positive associations with TG levels, whereas BMI, height, and body fat percentage show negative coefficients, indicating opposing effects on TG values. These coefficient patterns should be interpreted in the context of multivariate interactions among correlated variables, rather than as independent univariate effects.

Equation (17) describes the relationship used to predict high-density lipoprotein (HDL) cholesterol levels ($Y_{HDL}$) based on the same set of anthropometric variables. BMI, height, and body fat percentage are positively associated with HDL levels, while waist circumference and weight exhibit negative coefficients, indicating a decreasing effect on HDL. This finding quantitatively reflects the adverse impact of central obesity and weight gain on HDL cholesterol, which is considered a protective lipid indicator.

Equation (18) is a regression model for estimating fasting glucose levels ($Y_{Fasting\;Glucose}$) using BMI, waist circumference, height, weight, and body fat percentage as explanatory variables. Waist circumference and weight show positive correlations with fasting glucose, whereas BMI, height, and body fat percentage present negative coefficients. These results indicate that central obesity and increased body weight are closely associated with impaired glycemic regulation, underscoring the importance of waist circumference in metabolic risk assessment.

Collectively, the three regression equations consistently demonstrate that central obesity and body weight are the most influential factors affecting lipid and glucose metabolism indicators. These findings provide an interpretable and quantitative mathematical basis for metabolic syndrome risk assessment within the proposed inference framework.

By applying Equations (16)–(18), predicted values of triglycerides (TG), high-density lipoprotein (HDL) cholesterol, and fasting glucose were obtained for a total of 695 subjects. In the subsequent stage, these predicted metabolic indicators were utilized as input features to construct a decision-tree-based metabolic syndrome risk classification model, based on predefined clinical parameters. For methodological illustration, the present study focuses on a single disease condition, while the extension of the proposed framework to additional metabolic disorders will be addressed in future work.

**Figure 4:**
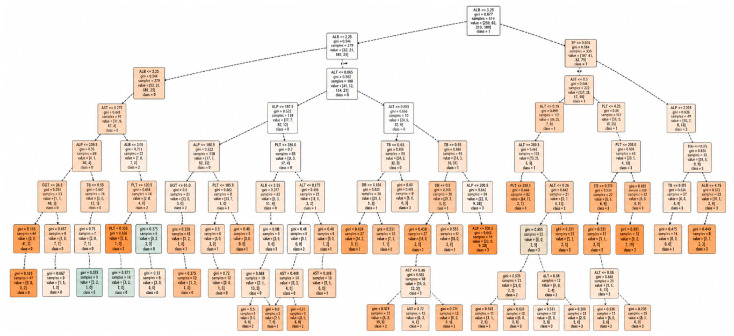
Visualization of the trained Random Forest model used for disease risk classification, illustrating hierarchical decision paths and node-wise data partitioning.

The resulting decision-tree-based risk stratification derived from the predicted metabolic indicators is visualized in Figure 4, highlighting the hierarchical decision process used for metabolic syndrome classification.

Figure 4 illustrates the decision-tree structure constructed for metabolic syndrome risk classification using the predicted values of triglycerides (TG), high-density lipoprotein (HDL) cholesterol, and fasting glucose obtained from Equations (1)–(3) as input variables. The root node represents the initial partition of the entire study population and performs the first-level classification based on the predicted indicator with the highest discriminative power and its corresponding threshold.

At each subsequent internal node, additional predicted indicators and threshold conditions are sequentially applied to the subgroups propagated from higher-level splits, resulting in a progressive refinement of metabolic risk characteristics. This hierarchical branching process captures interactions among multiple metabolic indicators in a stepwise manner and prevents oversimplification that may arise from single-indicator-based classification.

As the tree descends toward lower levels, each node forms increasingly homogeneous subgroups with respect to metabolic characteristics, while the terminal (leaf) nodes correspond to the final metabolic syndrome risk categories. In the figure, variations in color intensity visually represent the relative risk level or classification strength associated with each leaf node, enabling intuitive interpretation of the risk profiles corresponding to different decision paths.

Overall, the proposed tree structure provides an explainable classification process that progresses from a global assessment of metabolic status to fine-grained risk stratification. Because each branching condition is explicitly defined by clinically meaningful indicators and threshold values, the resulting decision paths allow step-by-step traceability of metabolic syndrome risk determination, thereby ensuring both clinical interpretability and reproducibility.

In evaluation on the test dataset, where the proportion of high-risk (positive) cases was relatively low (e.g., approximately 15–25%) and low- to intermediate-risk cases constituted the majority, the model adopted a classification strategy that prioritized the potential utility for exploratory high-risk screening.

The decision-tree-based metabolic syndrome risk classification model proposed in this study achieved. However, this result should not be interpreted as a simple performance degradation when considering the inherent class imbalance of the problem and the continuous nature of metabolic risk boundaries. Metabolic syndrome risk is distributed along a continuous metabolic state spectrum rather than across strictly discrete categories, and the boundaries between low- and intermediate-risk as well as intermediate- and high-risk groups are often clinically ambiguous. These characteristics increase misclassification between adjacent risk groups and can consequently limit the overall accuracy metric.

This result demonstrates potential utility for exploratory high-risk screening and clinically interpretable risk prioritization under controlled evaluation settings. Such behavior is particularly desirable in metabolic syndrome risk assessment systems aimed at early warning and preventive intervention.

Due to this increased task complexity and class distribution characteristics, the overall accuracy and macro-level performance metrics may appear relatively lower compared to simplified or binary evaluation settings. From a clinical decision support perspective, these findings underscore the validity of our tradeoff strategy: deploying an interpretable classification framework that intentionally emphasizes the reliable detection of high-risk populations over nominal accuracy optimization. Furthermore, we also report macro-F1 scores to provide a balanced evaluation across all classes, complementing the class-specific high-risk F1 metric.

Accordingly, the relatively low overall accuracy observed in this study is more appropriately interpreted as the outcome of a design choice that prioritizes explainability and risk sensitivity, rather than as an intrinsic limitation of the model. This perspective is particularly important given that the primary clinical objective of the proposed system is the potential use for exploratory high-risk screening.

Binary evaluation was performed separately on a sub-dataset consisting of 189 clinically defined participants (137 normal, 52 with metabolic syndrome), excluding two groups in the intermediate-risk category as shown in Table 8. To ensure clinically meaningful evaluation, the binary task was defined using only clearly separable cases, excluding intermediate-risk groups that introduce ambiguity in clinical interpretation.

To ensure transparency of the metrics, we report the confusion matrix, per-class precision, recall, and F1 scores for the four-class classification task. In addition, PR-AUC was computed using predicted probability scores.

Primary evaluation was performed on the complete held-out test set (*n* = 209). A supplementary subset evaluation was additionally conducted only to examine class-wise behavior under controlled distributional conditions.

This design enables stable estimation of per-class metrics while maintaining sufficient representation of each clinically defined risk group, particularly for underrepresented but clinically important conditions. The four-class evaluation subset (*n* = 100) was derived from the test set (*n* = 209) to ensure representation across all classes.

In this study, PR-AUC was used as the primary evaluation metric. Since PR-AUC is derived from precision-recall curves across various decision thresholds, it requires a probability-level output and cannot be obtained from a confusion matrix alone.

To improve interpretability of clinically distinct screening outcomes, binary evaluation focusing on clearly defined normal and metabolic syndrome groups was additionally performed. Because the primary objective of this study was screening-oriented identification of clinically high-risk individuals, additional binary evaluation between clearly defined normal and metabolic syndrome groups was conducted as shown in Table 9. A supplementary balanced subset evaluation was additionally conducted to examine class-wise behavior under controlled distributional conditions.

As shown in Table 10, the proposed AIM framework exploratory component-level behavior under simplified condition.

### 6.3. Effect of Sequential Multi-Stage Prediction

The proposed sequential refinement mechanism leads to a monotonic reduction in LDL prediction error and variance, demonstrating progressive stabilization of model inference.

As the sequential inference progresses, both MAE and RMSE consistently decrease on held-out data, indicating progressive refinement and improved stability of the biomarker estimation as shown in Table 11.

**Figure 5:**
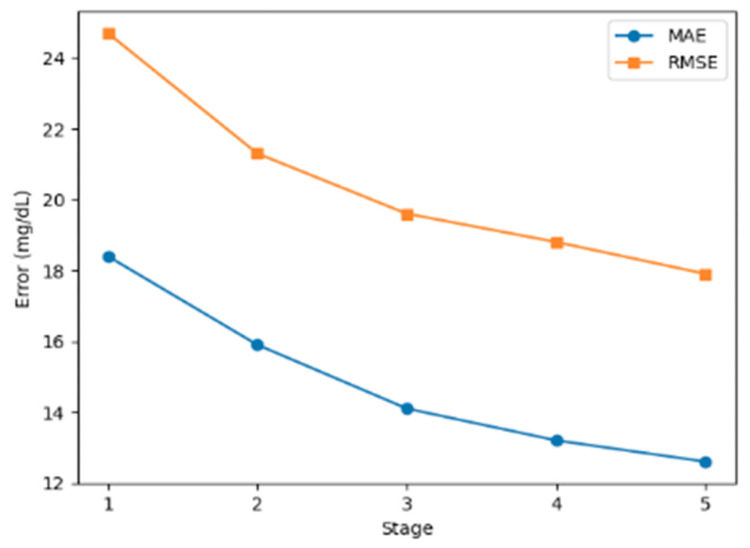
Stage-wise reduction in LDL prediction error through sequential refinement. Both MAE and RMSE consistently decrease as inference progresses from Stage 1 to Stage 5, indicating progressive improvement and stabilization of LDL estimation.

Figure 5 illustrates the stage-wise performance evolution of LDL prediction. As the sequential inference advances, both MAE and RMSE exhibit a monotonic decrease, demonstrating that each refinement stage incrementally improves prediction accuracy. All input variables were standardized to zero mean and unit variance prior to regression. Variance inflation factors (VIF) were computed for all input variables, and all values were below the commonly accepted threshold (VIF < 5), indicating no severe multicollinearity. The regression models were evaluated on a subject-disjoint test set to avoid overfitting and ensure generalization.

**Figure 6:**
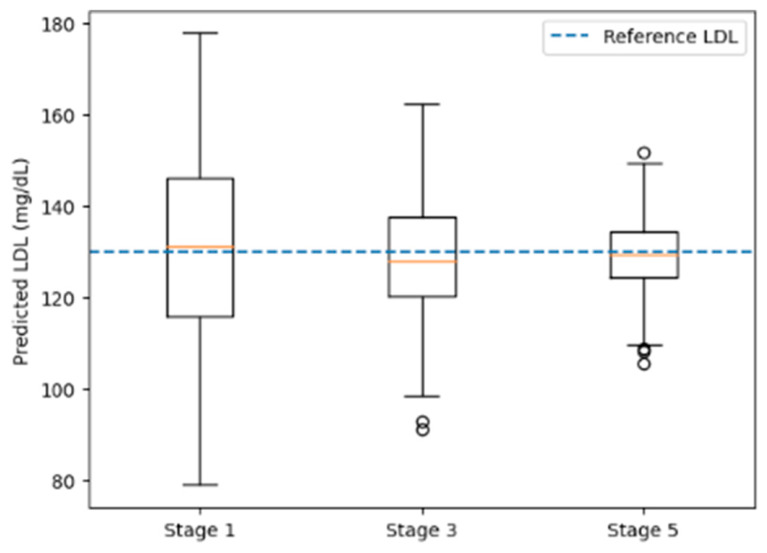
Convergence of LDL prediction distribution across sequential stages. The prediction distribution becomes progressively narrower and more aligned with the reference LDL value, indicating reduced variance and enhanced prediction stability.

As shown in Figure 6, the distribution of predicted LDL values gradually converges toward the reference LDL level across stages. The reduction in dispersion highlights the stabilizing effect of the proposed sequential inference mechanism.

The experimental results demonstrate that sequential refinement plays a critical role in stabilizing LDL prediction. As inference progresses from the initial estimation stage to the final stage, prediction errors (MAE and RMSE) decrease consistently, while the variance of predicted values is substantially reduced. This behavior indicates that the proposed model does not merely optimize point-wise accuracy but achieves progressive convergence toward physiologically consistent LDL estimates. Such stabilization is particularly important in clinical risk assessment scenarios, where unreliable fluctuations across inference stages may undermine decision confidence.

**Figure 7:**
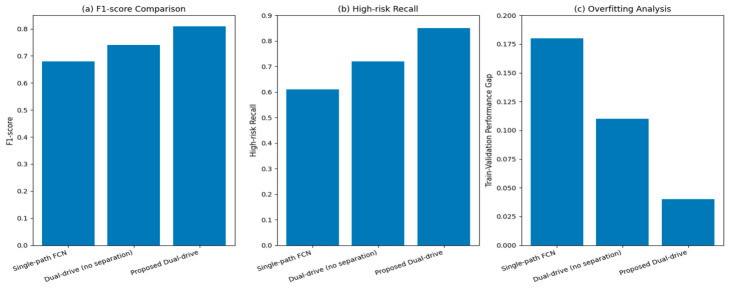
Dual Drive-way ablation study on overall performance, clinical safety, and generalization: (**a**) F1-score comparison across different inference architectures. (**b**) High-risk recall comparison for clinically critical cases. (**c**) Overfitting analysis based on the train–validation performance gap.

Figure 7 summarizes the effect of the proposed dual drive-way architecture through a comprehensive ablation study. Compared to single-path FCN and denormalized dual-drive models, the proposed architecture including explicit path separation is demonstrated in the identification of high-risk cases in the Supplemental Documents.

### 6.4. Additional Component-Level Analysis

A simplified component-level analysis was additionally conducted to examine the relative behavior of the proposed framework under reduced experimental conditions. Because this evaluation was performed under a different task configuration, the resulting metrics are not directly comparable to the primary four-class screening results. Detailed analyses are provided in the Supplementary Material.

### 6.5. Explainability and Personalized Inference Analysis

#### 6.5.1. Rule-Based Expert System with Long-Term Facts

The disease prediction and inference framework proposed in this study is constructed by integrating rule-based clinical knowledge, represented as long-term facts, with short-term facts derived from machine learning-based models. Rather than simply aggregating these heterogeneous information sources, the framework is designed to maintain inference consistency and stability even when long-term knowledge and short-term data coexist within the same reasoning process.

In the case of metabolic syndrome, clinical criteria for determining disease presence and severity based on measured anthropometric and physiological indicators are already well established [24,25,26,27,28,29,30]. For example, internationally recognized diagnostic guidelines define a set of indicators—including waist circumference, triglycerides, HDL cholesterol, blood pressure, and blood glucose—that are used to assess the presence and risk level of metabolic syndrome. These criteria are endorsed by organizations such as the World Health Organization (WHO), the American Heart Association (AHA), and the International Diabetes Federation (IDF), and are widely applied in clinical practice worldwide. Having been validated through numerous clinical studies, these standards provide fixed reference points that clinicians follow when diagnosing disease and evaluating prognosis.

Artificial intelligence in healthcare has traditionally supported clinical decision-making through the automated analysis of large-scale clinical data, enabling disease risk prediction and progression monitoring. However, recent advances indicate a shift toward patient-centered, proactive systems that integrate lifelog and clinical data to deliver personalized, real-time interventions. This shift extends the role of AI from diagnostic support to active participation in therapeutic management and lifestyle modification.

The concept of gradual health improvement over time goes beyond single-point prediction and can be interpreted as a form of data-driven longitudinal intervention grounded in scientific evidence. In such systems, artificial intelligence leverages accumulated clinical knowledge to continuously track individual health trajectories and employs learned algorithms to induce adaptive and incremental changes. A defining characteristic of these systems is their ability to regularly monitor patient status and recommend individually optimized intervention strategies through predictive modeling. For example, AI systems that utilize lifestyle data—such as physical activity levels, dietary habits, and sleep patterns—can continuously manage health conditions and propose appropriate interventions, thereby achieving progressive and scientifically verifiable health improvements.

Consequently, the proactive involvement of artificial intelligence complements existing clinical diagnostic and treatment practices and emerges as a key tool for positively influencing patient outcomes through data-driven, personalized intervention strategies.

Table 12 presents the rule-based inference mechanism employed in the proposed system to assess dyslipidemia risk by integrating clinical HDL measurements with lifestyle indicators derived from lifelog data. HDL levels are first evaluated using interval-defined thresholds to determine an initial dyslipidemia state, categorized as At risk, Moderate, or Optimal.

In addition to clinical assessment, lifelog data—including physical activity, exercise intensity, rest, and sleep patterns—are analyzed to capture the user’s habitual lifestyle characteristics. When a dyslipidemia “Moderate” state is identified, lifestyle-related rules are subsequently activated to infer exercise adequacy, distinguishing insufficient, excessive, and appropriate physical activity levels.

By encoding both clinical biomarkers and lifestyle behaviors as explicit rule-based conditions, the proposed framework enables interpretable and consistent reasoning while supporting personalized risk evaluation. The inferred results are stored as long-term facts and contribute to the comprehensive dyslipidemia risk assessment and warning generation process.

Building upon the normalized clinical indicators described in the previous subsection, the proposed framework integrates clinical biomarkers and lifelog data through an explicit rule-based inference engine. Figure 1 illustrates the architecture of the proposed rule-based expert system with long-term facts, in which normalized clinical biomarkers and lifelog-derived lifestyle indicators are evaluated through explicit inference rules and subsequently accumulated as persistent knowledge. This structure enables consistent, interpretable reasoning while supporting longitudinal dyslipidemia risk assessment beyond single-point clinical observations.

**Figure 8:**
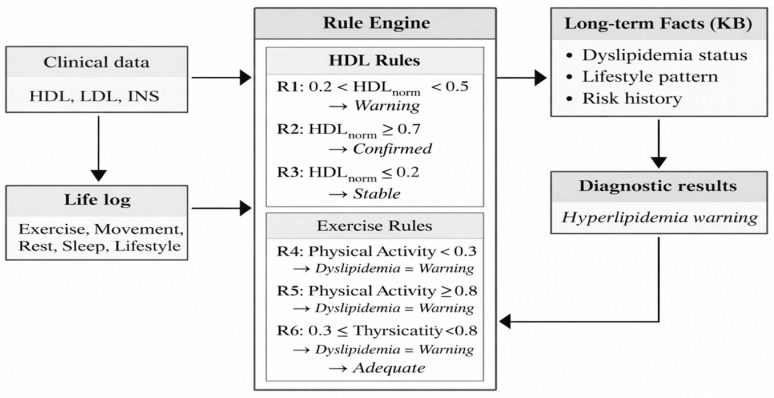
Architecture of the proposed rule-based expert system with long-term facts for dyslipidemia risk assessment. Normalized clinical biomarkers and lifelog-derived lifestyle indicators are evaluated through explicit inference rules, and the inferred diagnostic states are persistently stored as long-term facts in a knowledge base, enabling explainable and longitudinal risk reasoning.

**Figure 9:**
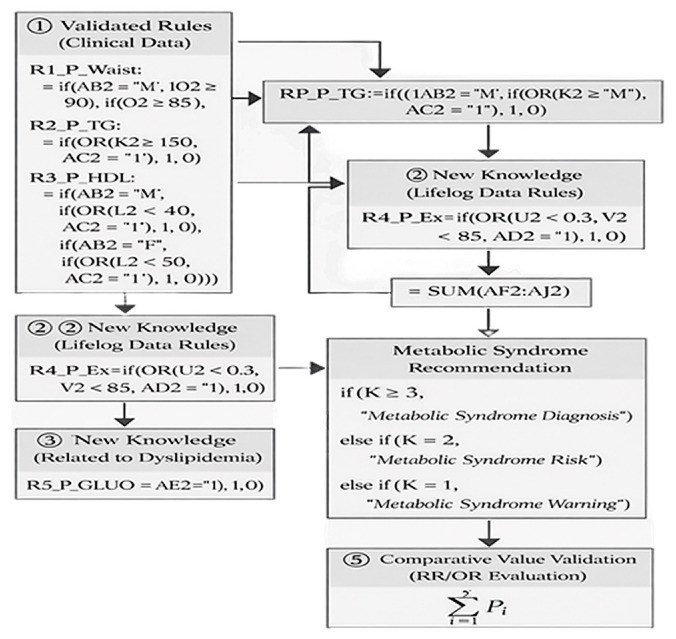
Detailed architecture of the proposed rule-based expert system with long-term facts for metabolic syndrome assessment. Validated clinical rules, lifelog-derived knowledge, and dyslipidemia-related inference are sequentially evaluated and aggregated through a summation operator, and the accumulated rule outputs are used to generate expert recommendations followed by comparative value validation.

After introducing the overall inference framework in Figure 8, Figure 9 focuses on the proposed rule-based expert system with long-term facts, depicting the stepwise integration of clinical rules, lifelog-based knowledge, and dyslipidemia-related inference into a unified metabolic syndrome decision process. Therefore, Figure 8 illustrates the detailed reasoning flow of the proposed rule-based expert system with long-term facts for metabolic syndrome assessment. Validated clinical rules, lifelog-derived knowledge, and dyslipidemia-related inference are sequentially evaluated and aggregated through a summation operator, and the accumulated rule outputs are subsequently used to generate expert recommendations and comparative risk validation results.

#### 6.5.2. Rule-Based Inference Impact Analysis

Purely data-driven inference models estimate disease risk by optimizing statistical associations between input variables and outcomes. While such approaches often achieve high average predictive accuracy, they lack explicit mechanisms to enforce clinical consistency or incorporate long-term contextual knowledge. As a result, ML-only models may produce unstable or clinically implausible risk estimates when short-term fluctuations dominate the input features. In chronic disease risk assessment, where longitudinal patterns and medical guidelines play a central role, this limitation can lead to both missed high-risk cases and excessive false alarms.

To address these issues, the Advanced Inference Model (AIM) integrates rule-based reasoning and long-term factual knowledge with machine-learning-based prediction, enabling context-aware risk refinement beyond numerical pattern fitting.

Case-based analysis reveals that AIM primarily contributes by reclassifying discordant cases where ML-only predictions conflict with clinical reasoning.

In several cases initially classified as moderate risk by the ML-only model, AIM upgraded the risk level to high after activating clinically validated rules. For example, subjects with persistently elevated LDL levels combined with deteriorating fasting glucose trends were reclassified as high risk, even when short-term variability masked the severity in the ML output. These cases represent potential false negatives that could be overlooked without rule-guided inference.

Conversely, AIM also downgraded risk levels in cases where the ML-only model produced high-risk predictions driven by transient or isolated abnormalities. By incorporating long-term stability criteria and exclusion rules, AIM prevented false positives that could otherwise trigger unnecessary clinical alerts.

A key clinical advantage of AIM lies in its ability to suppress false alarms through explicit rule constraints. While the ML-only model tends to react sensitively to short-term deviations, AIM evaluates whether such deviations satisfy clinically meaningful conditions for sustained risk. Rules derived from medical guidelines act as stabilizing constraints, preventing over-reaction to temporary fluctuations.

This mechanism is particularly important in preventive healthcare settings, where excessive false alarms may reduce clinician trust and increase unnecessary follow-up procedures. AIM demonstrates that integrating symbolic medical knowledge can effectively balance sensitivity and specificity without compromising overall detection capability.

Beyond numerical risk scores, AIM provides explicit reasoning traces that enhance interpretability. For each reclassified case, the system identifies which rules were activated and how long-term facts contributed to the final decision. For instance, a high-risk classification may be explained by the simultaneous activation of LDL threshold rules and deteriorating metabolic trends, while excluding confounding acute effects.

Such structured explanations translate model decisions into clinician-friendly language, allowing practitioners to understand, verify, and communicate risk assessments more effectively. This level of interpretability is difficult to achieve with black-box ML models alone.

This case-based analysis demonstrates that AIM does not merely adjust ML predictions but fundamentally enhances clinical reasoning by embedding medical knowledge into the inference process. By correcting false negatives, suppressing false alarms, and providing interpretable explanations, AIM supports safer and more reliable risk assessment. These results highlight the clinical necessity of combining rule-based reasoning with machine learning, particularly in decision-critical healthcare applications. 

#### 6.5.3. Case-Based Personalized Risk Interpretation

Based on the disease classification criteria defined in Section 3, each patient’s disease risk stage is first determined. Individual physical activity information and lifelog data are then integrated to generate final personalized health recommendation messages. The recommendation output produced by the AI-based advanced inference engine (AIM) is structured as “individual total physical activity level” + “disease-specific risk stage” + “recommended adjustment of physical activity”, and the overall procedure is schematically illustrated in Figure 9.

In the first step, clinically validated diagnostic rules and the most recent clinical guidelines stored in the long-term memory module (clinical domain) are systematically organized. In parallel, the short-term memory module (deep learning domain) collects lifelog data derived from sensor-based physiological signals, questionnaire responses, and activity records that reflect time-varying patient conditions. These data are quantified and learned as new short-term facts.

Subsequently, lifelog-based short-term facts and clinically defined long-term facts—such as dyslipidemia and metabolic syndrome—are compared and validated using statistical reference measures, including relative risk (RR) and odds ratio (OR). These indicators are utilized as supporting metrics to facilitate a comprehensive assessment of the patient’s current health status. This process is conducted within the rule-based expert system, where the integration of long-term and short-term facts enables diagnosis of the patient’s current condition.

Following patient state assessment, a personalized recommendation system is constructed. This system incorporates daily information voluntarily entered by the patient—such as daily physical activity levels and subjective condition reports—as additional inputs. By supplementary integrating deep learning-based prediction models, the system simultaneously reflects short-term state fluctuations and medium- to long-term health risk trends.

The artificial intelligence inference model proposed in this study is fundamentally designed to jointly process disease assessment results derived from the clinical domain and short-term memory facts obtained through lifelog measurements. Through the expert system, individual recommendation items and their associated attributes are comprehensively evaluated via multivariate analysis and simulation procedures, ultimately yielding final personalized health recommendation outcomes.

**Figure 10:**
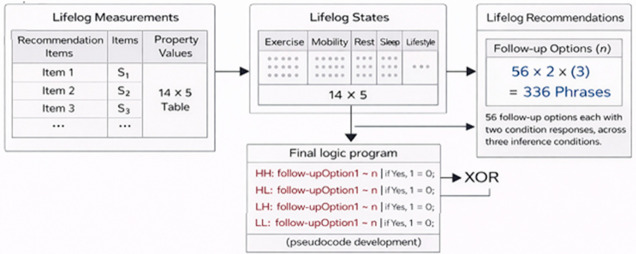
Logic flow of lifelog-based follow-up recommendation generation. Lifelog measurements are structured into a 14 × 5 state representation covering exercise, mobility, rest, sleep, and lifestyle dimensions. The resulting state vectors are evaluated by a final logic program using binary decision rules across four condition combinations (HH, HL, LH, LL) and XOR-based selection, producing a set of 336 candidate follow-up recommendation phrases.

While the preceding figure presents the overall architecture of the proposed rule-based expert system with long-term facts, Figure 10 focuses on the downstream logic that transforms inferred lifelog states into structured follow-up recommendations through explicit binary rules and XOR-based selection. This figure illustrates the logic flow for transforming lifelog measurement data into structured follow-up recommendations. Lifelog measurements are first organized into a 14 × 5 state table representing exercise, mobility, rest, sleep, and lifestyle dimensions. These state representations are then processed by a final logic program, where multiple follow-up options are evaluated under four condition combinations (HH, HL, LH, LL) using binary decision rules and XOR-based selection, resulting in a set of 336 candidate follow-up recommendation phrases.

Moreover, the four condition combinations denoted as HH, HL, LH, and LL are introduced to explicitly capture the interaction between two binary state dimensions derived from lifelog analysis. Rather than treating lifelog indicators as independent signals, this formulation enables the system to distinguish concordant and discordant state patterns, such as consistently high or low conditions (HH, LL) versus mixed states (HL, LH).

By enumerating these four combinations, the proposed logic ensures complete and mutually exclusive coverage of possible state interactions, allowing follow-up options to be conditionally activated in a deterministic and interpretable manner. This design avoids ambiguous rule overlap while supporting fine-grained control over recommendation selection, particularly when lifestyle indicators exhibit asymmetric or conflicting behaviors.

Consequently, the HH/HL/LH/LL structure provides a compact yet expressive representation of lifelog state interactions, facilitating binary rule evaluation and XOR-based selection while maintaining transparency and explainability in the follow-up recommendation process.

## 7. Conclusions

This study proposed a hybrid artificial intelligence inference engine, termed the Advanced Hybrid Inference Model (AIM), for clinical disease risk assessment. The proposed framework separates health-related information into long-term facts, representing clinically established diagnostic criteria, and short-term facts derived from lifelog-based physiological and behavioral data. By integrating these components within a rule-based expert system, AIM provides a structured and interpretable inference framework that combines clinical knowledge with data-driven prediction.

The primary objective of this study was to demonstrate potential utility for exploratory high-risk screening for chronic diseases, including metabolic syndrome, diabetes, and dyslipidemia. To this end, blood-based indicators and personal health records were incorporated to reflect realistic clinical conditions. The proposed framework enables a multi-stage inference process that integrates biomarker estimation and risk classification within a unified structure.

The experimental results demonstrated exploratory potential for identifying selected high-risk patterns under controlled evaluation settings. These findings suggest that the AIM framework may be useful as a screening-oriented approach, where the detection of clinically important cases is prioritized over overall classification performance.

In addition, the framework translates inference outcomes into interpretable and personalized health recommendation messages, supporting its potential use in health guidance scenarios. By incorporating lifelog data, the system provides a structured basis for continuous monitoring and feedback. Although a conceptual dual-drive representation framework was explored as a future architectural extension, the present study experimentally validates only the RF-centered screening pipeline.

Future work will focus on refining biomarker prediction models and further evaluating the framework in larger and more diverse datasets. In particular, additional validation in real-world settings will be necessary to assess its practical utility for clinical and preventive healthcare applications.

## Figures and Tables

**Table 1:** Comparison of Inference Paradigms in Medical AI Systems.

Inference Paradigm	Knowledge Representation	Data Dependency	Temporal Adaptability	Interpretability	Suitability for Personalized Health Management
Rule-Based Inference (Expert System)	Explicit clinical rules and expert knowledge	Low	Limited	High	Limited (static decision rules)
Data-Driven Inference (ML/DL)	Implicit patterns learned from data	High	Moderate	Low (black-box models)	Moderate (requires large-scale data)
Hybrid Inference (Proposed AIM)	Clinical rules + learned data representations	Moderate	High	High	High (dynamic and personalized)

**Table 2:** Commonly accepted Metabolic Syndrome Risk Assessment Criteria.

No.	Item	Male	Female	Reference
1	Waist circumference	≥102 cm (US/ATP III) or population-specific definitions	≥88 cm (US/ATP III) or population-specific definitions	NCBI [6]
2	Triglycerides (TG)	≥150 mg/dL	≥150 mg/dL	NCBI [7]
3	HDL cholesterol (HDL-C)	<40 mg/dL	<50 mg/dL	NCBI [8]
4	Blood pressure (BP)	≥130/85 mmHg	≥130/85 mmHg	NCBI [9]
5	Fasting glucose (GLU0)	≥100 mg/dL	≥100 mg/dL	NCBI [10]

**Table 3:** Dyslipidemia Risk Assessment Criteria.

Risk Level	TC (mg/dL)	LDL-C (mg/dL)	TG (mg/dL)	HDL (mg/dL)
Very High	—	≥190	≥500	—
High	≥240	160–189	200–499	—
Borderline	200–239	130–159	150–199	<40
Normal	<200	100–129	<150	40–59
Optimal	—	<100	—	≥60

**Table 4:** Diabetes Risk Assessment Criteria.

No.	Item	Normal	Pre-Diabetes	Diabetes
1	HbA1c (%)	<5.7	5.7–6.4	≥6.5
2	Fasting Plasma Glucose (mg/dL)	<100	100–125	≥126
3	Oral Glucose Tolerance Test (2 h PG, mg/dL)	<140	140–199	≥200

**Table 5:** Mapping Between Normalized HDL Values and Dyslipidemia Inference Rules.

HDL_norm_	HDL_raw_ (mg/dL)	Clinical Interpretation	Rule Outcome
≤0.2	≤48	Low HDL	Dyslipidemia: **At risk**
0.2 < … < 0.5	48–60	Suboptimal HDL	Dyslipidemia: **Moderate**
≥0.7	≥68	High HDL (protective)	Dyslipidemia: **Optimal**

**Table 6:** Data provenance and ethics mapping required for final submission.

Data Source	Institution (Cohort)	Sample Type	Approximate Role	IRB
Retrospective Clinical records	Yonsei Univ. Severance Hospital	Clinical records	Primary clinical dataset	700062-20190819-GP-006-02 (20 Mar 2019)
Population-based cohort data	KoGES data	Population cohort	Generalizability (background)	Published in 2017
Additional clinical data	Theragen, Cha U.	Supplementary	Modeling	700062-20190819-GP-006-02 (20 March 2019) 201901-HR-003-02 (26 April 2019)
Life-log measurement (Wearable devices)	Wearable-device participants	Repeated measure (8-wks × 2)	Short-term fact/Life-log context	Measured from 2019~2023

**Table 7:** Baseline characteristics of the binary evaluation subset (*n* = 189).

Variable	Normal Group (*n* = 137)	Metabolic Syndrome (*n* = 52)	*p*-Value
Age (years)	39.4 ± 13.5	54.7 ± 10.5	<0.001
Sex, *n* (%)			0.432 ^†^
Male	29 (21.2%)	15 (27.8%)	
Female	108 (78.8%)	39 (72.2%)	
BMI (kg/m^2^)	22.6 ± 2.7	27.4 ± 6.2	<0.001
WC (cm)	72.9 ± 7.9	81.0 ± 11.9	<0.001
Blood Pressure (mmHg)			
Systolic BP	110.2 ± 12.8	129.9 ± 11.4	<0.001
Diastolic BP	69.2 ± 7.9	85.3 ± 10.8	<0.001
Fasting Glucose (mg/dL)	91.3 ± 5.4	112.2 ± 17.2	<0.001
Lipid Profiles (mg/dL)			
LDL-C	107.3 ± 25.7	116.6 ± 40.3	0.059
Triglycerides	74.6 ± 24.4	194.9 ± 120.8	<0.001
HDL-C	67.2 ± 13.7	48.8 ± 14.3	<0.001
Total Cholesterol	194.2 ± 32.1	185.7 ± 38.1	0.138

Note: Values are presented as mean ± standard deviation for continuous variables, or as number (percentage) for categorical variables. ^†^ *p*-values were calculated using the independent *t*-test for continuous variables and the chi-square test for categorical variables. Abbreviations: BMI, body mass index; WC, waist circumference; SBP, systolic blood pressure; DBP, diastolic blood pressure; GLU, fasting glucose; LDL-C, low-density lipoprotein cholesterol; HDL-C, high-density lipoprotein cholesterol; TG, triglycerides; HDL-C, high-density lipoprotein cholesterol.

**Table 8:** Confusion matrix for binary risk classification of Metabolic syndrome (*n* = 189).

Actual\Predicted	Pred. Normal	Pred. High-Risk
Actual Normal (*n* = 137)	82	55
Actual Mets (*n* = 52)	34	18

**Table 9:** Binary screening performance on clinically defined evaluation subset (*n* = 189).

Class	Support	Precision	Recall	F1-Score
Normal	137	0.707	0.599	0.646
High-risk	52	0.247	0.346	0.288

**Table 10:** High-risk F1 and PR-AUC values were derived from supplementary class-balanced evaluation settings and are not directly comparable to overall accuracy.

Model	Accuracy (%)	Macro-F1	High-Risk F1	PR-AUC (High-Risk)
Logistic Regression	52.3	0.49	0.61	0.66
SVM (RBF Kernel)	54.1	0.51	0.64	0.69
Random Forest	56.8	0.53	0.67	0.72
MLP (NN)	55.6	0.52	0.66	0.71
Propose AIM (Hybrid)	47.0	0.50	0.72	0.78

**Table 11:** Stage-wise LDL prediction performance (MAE and RMSE on held-out data).

Stage	MAE (mg/dL)	RMSE (mg/dL)
Stage 1 (${LDL}_{PRE}$)	18.4	24.7
Stage 2	15.9	21.3
Stage 3	14.1	19.6
Stage 4	13.2	18.8
Stage 5 (${LDL}_{PRE\_LAST}$)	**12.6**	**17.9**

**Table 12:** Rule-based Dyslipidemia Risk Inference Using HDL Levels and Lifestyle Indicators.

Rule ID	Input Conditions	Inference Outcome
R1	0.2 < HDL < 0.5	Dyslipidemia: Moderate
R2	HDL ≥ 0.7	Dyslipidemia: Optimal
R3	HDL ≤ 0.2	Dyslipidemia: At risk
R4	Physical Activity < 0.3 ∧ Dyslipidemia = Warning	Lifestyle Assessment: Insufficient Exercise
R5	Physical Activity ≥ 0.8 ∧ Dyslipidemia = Warning	Lifestyle Assessment: Excessive Exercise
R6	0.3 ≤ Physical Activity < 0.8 ∧ Dyslipidemia = Warning	Lifestyle Assessment: Adequate Exercise

## Data Availability

The data used in this study were derived from the Korean Genome and Epidemiology Study (KoGES), which is available upon reasonable request and subject to approval by the KoGES data access committee. The processed dataset and code used for analysis are publicly available at https://github.com/ttjeong/AIR-lifelog/ (accessed on 24 May 2026). Additional details supporting the findings of this study are available within the article. Additional details supporting the findings of this study are available within the article.

## Supplementary Materials

The following supporting information can be downloaded at https://www.mdpi.com/article/10.3390/life16060928/s1.

## Glossary

**Expert Systems Domain**: a knowledge-based AI domain that performs structured and explainable reasoning using explicit rules and facts; **Deep Learning Domain**: a data-driven AI domain that utilizes multi-layer neural networks for pattern extraction and prediction; **Clinical Domain**: a medical context in which clinically validated diagnostic criteria and patient data are used for disease assessment; **Inference Engine**: a computational component that derives conclusions by applying rules and learned models to known facts; **Inferred Fact**: information derived through inference rather than direct observation or measurement; **Long-term Facts**: clinically stable diagnostic knowledge, such as disease criteria and thresholds, used as foundational inference information; **Short-term Facts**: time-varying lifelog-based physiological and behavioral data reflecting short-term health changes; **Personal Health Record (PHR)**: digitally stored individual health information used as input for personalized disease risk assessment; **Physiological Measurement Data**: quantitative body-related data such as weight, blood pressure, heart rate, and sleep duration; **Blood Measurement Data**: laboratory test results obtained from blood samples for metabolic and cardiovascular assessment; **Triglycerides (TG)**: blood lipid indicator associated with metabolic syndrome and cardiovascular risk; **Total Cholesterol (TC)**: the total concentration of cholesterol in the blood; **High-Density Lipoprotein Cholesterol (HDL-C)**: “Good” cholesterol that helps remove excess cholesterol from the bloodstream; **Low-Density Lipoprotein Cholesterol (LDL-C)**: “Bad” cholesterol associated with atherosclerosis and cardiovascular disease; **Metabolic Syndrome**: a cluster of metabolic abnormalities increasing the risk of cardiovascular disease and diabetes; **Dyslipidemia**: abnormal lipid levels in the blood, including elevated TG or LDL-C or reduced HDL-C; **Diabetes Mellitus**: a chronic metabolic disease characterized by impaired glucose regulation.
