# Supplementary material for: AIM: An Advanced Hybrid Inference Model Combining Clinical Rules and Lifelog-Based Learning for Health Risk Prediction
Source: Life (Basel). 2026 Jun 1;16(6):928. doi: 10.3390/life16060928 (PMC13302408; doi:10.3390/life16060928)
Supplement: Supplementary file 1 [file life-16-00928-s001.zip › Supplimental Documents_S1.pdf]

Supplemental Documents #S1

This supplementary document provides an extended description of the expert system architecture introduced in Section 5 of the main text.

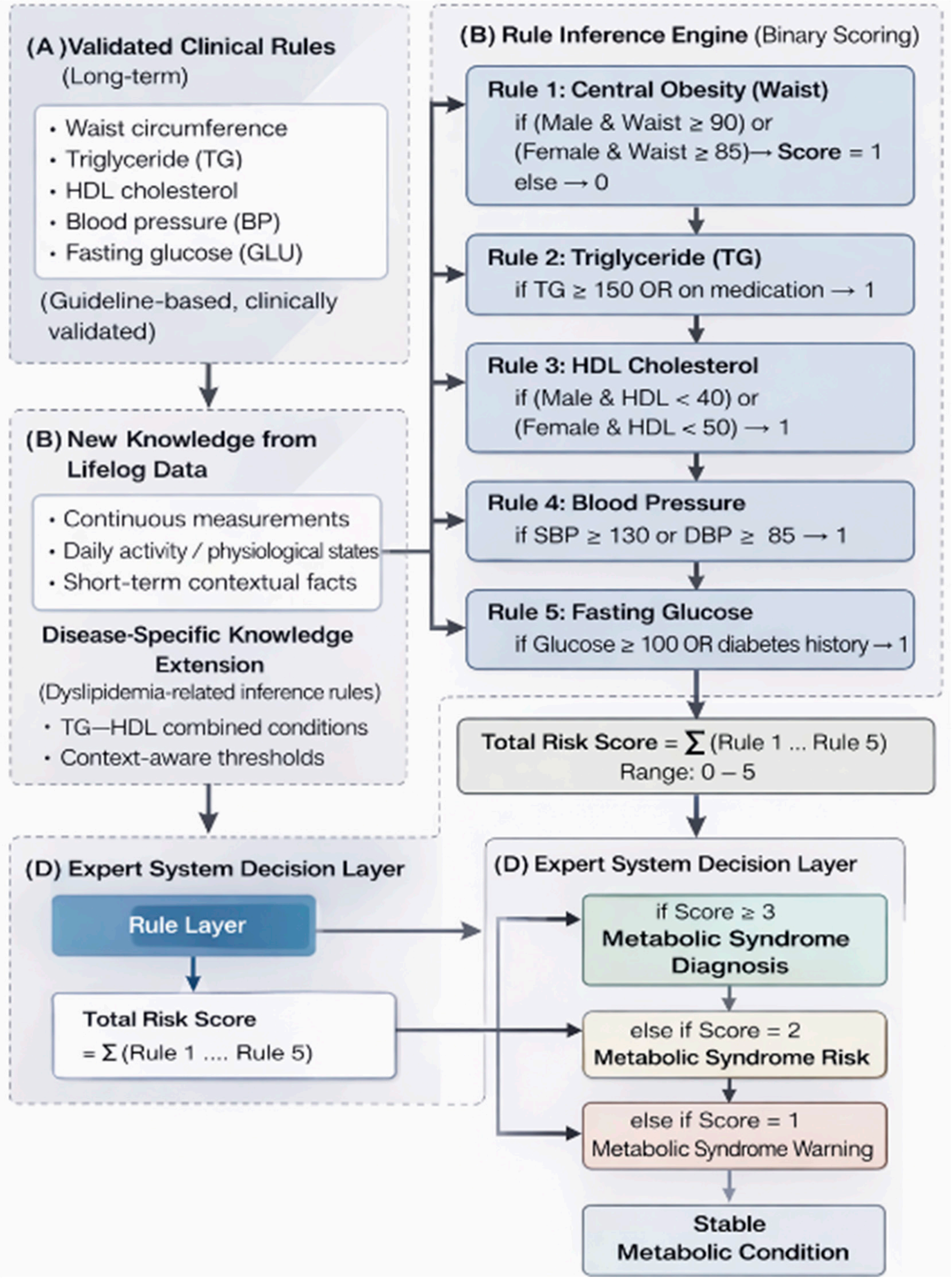

Figure-S1. Illustrates the overall architecture of a rule-based expert system for metabolic syndrome risk assessment, integrating clinically validated long-term rules with lifelog-derived short-term facts to generate

interpretable risk scores and expert-level diagnostic recommendations.

Figure-S1 illustrates the overall inference flow of the rule-based expert system designed for metabolic syndrome risk assessment. The system begins with a layered knowledge structure that explicitly separates clinically validated long-term facts from lifelog-derived short-term facts.

The long-term knowledge layer consists of core diagnostic indicators for metabolic syndrome defined by established clinical guidelines, including waist circumference, triglycerides, HDL cholesterol, blood pressure, and fasting glucose. In contrast, the short-term knowledge layer reflects dynamically changing facts derived from lifelog data, such as continuous physiological signal measurements, daily activity records, and short-term physiological states.

In addition, disease-specific knowledge expansion related to dyslipidemia is incorporated, allowing composite conditions and context-aware thresholds for triglycerides and HDL cholesterol to be considered during inference.

Each clinical indicator is subsequently evaluated as an independent binary rule by the rule inference engine. For central obesity, triglyceride level, HDL cholesterol, blood pressure, and fasting glucose, each rule outputs a value of 1 if the condition is satisfied and 0 otherwise. This binary structure enables full traceability of the decision-making process and supports explainable inference. The outputs of individual rules are aggregated additively to form a total risk score, defined over a range from 0 to 5.

Finally, within the expert system decision layer, the aggregated risk score is used to classify metabolic syndrome status in a stepwise manner. A total score of 3 or higher indicates metabolic syndrome, a score of 2 corresponds to a high-risk state, and a score of 1 is interpreted as a warning stage. If none of the criteria are met, the individual is classified as having a stable metabolic condition. This multi-level risk stratification framework extends beyond binary diagnosis and enables fine-grained, interpretable risk assessment suitable for clinical decision support systems.

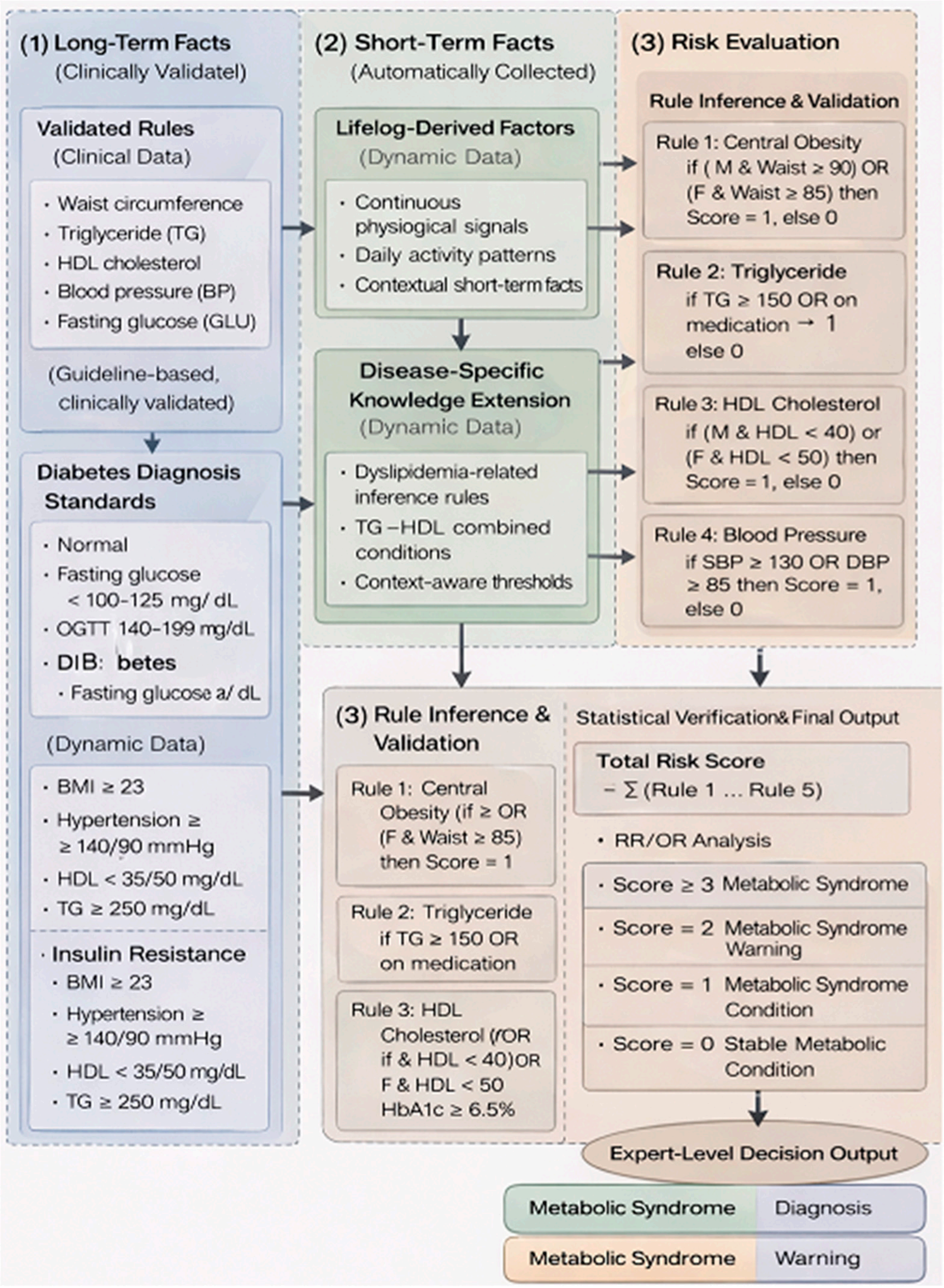

Figure-S2. Knowledge-Driven Expert System Architecture for Metabolic Syndrome and Diabetes Risk Assessment

Figure-S2 presents the overall architecture of the knowledge-driven expert system for assessing metabolic syndrome and diabetes risk. The system is designed with separate knowledge layers for long-term facts, derived from clinically validated data, and short-term facts, automatically collected from lifelog sources. These layers are integrated to perform rule-based inference and final risk assessment, enabling simultaneous consideration of stable clinical knowledge and time-varying behavioral and physiological changes.

The left-hand side represents the long-term fact layer, which includes key clinical examination indicators such as waist circumference, triglycerides, HDL cholesterol, blood pressure, and fasting glucose. This layer is structured around validated clinical rules defined by established guidelines. In addition, diagnostic criteria for diabetes—categorized into normal glucose tolerance, impaired fasting glucose, and diabetes—are incorporated together with major type 2 diabetes risk factors, including BMI, hypertension, dyslipidemia, and insulin resistance. These elements collectively provide a stable representation of an individual's baseline metabolic condition.

The central region corresponds to the short-term fact layer, which is constructed from lifelog data such as continuous physiological signals, daily activity patterns, and context-aware short-term information. Within this layer, disease-specific knowledge expansion for dyslipidemia is applied, enabling dynamic consideration of composite conditions and context-sensitive thresholds for triglycerides and HDL cholesterol. Through this mechanism, time-dependent physiological and behavioral variations are reflected in the risk assessment process, allowing personalized inference beyond rigid threshold-based diagnosis.

The right-hand side depicts the rule inference and validation stage. Each clinical indicator is evaluated using independent binary rules, where conditions related to central obesity, triglycerides, HDL cholesterol, blood pressure, and fasting glucose produce binary outputs (0 or 1). All rule outputs are aggregated additively to form a total risk score. In addition, statistical validation using relative risk (RR) and odds ratio (OR) is employed as supporting evidence to enhance the reliability and quantitative validity of the rule-based inference.

As shown in Fig. 4, the explicit separation and integration of long-term and short-term facts provide a generalized knowledge-based inference framework applicable not only to metabolic syndrome and diabetes but also to a wide range of chronic diseases. Based on this concept, the present study proposes an initial prototype of an expert system that integrates multiple objective fact layers with artificial intelligence–based inference models. This approach moves beyond conventional population-level criteria and establishes a foundation for personalized risk prediction and intervention by accounting for individual-specific physiological and behavioral dynamics.

Given that disease risk prediction is closely linked to patient prognosis, accurate assessment and appropriate intervention recommendations extend beyond traditional clinical workflows toward AI-driven precision medicine. Accordingly, the proposed system is realized as an advanced hybrid inference engine specialized for metabolic disorders—termed the Advanced Inference Model for Metabolism (AIM-M)—and demonstrates the potential of an explainable, scalable, next-generation platform for chronic disease risk assessment and personalized health management.
