# Supplementary material for: AIM: An Advanced Hybrid Inference Model Combining Clinical Rules and Lifelog-Based Learning for Health Risk Prediction
Source: Life (Basel). 2026 Jun 1;16(6):928. doi: 10.3390/life16060928 (PMC13302408; doi:10.3390/life16060928)
Supplement: Supplementary file 1 [file life-16-00928-s001.zip › Supplimental Documents_S2.pdf]

Supplemental Documents #S2

This supplementary document provides an extended description of the expert system architecture introduced in **Section 5** of the main text.

5.1. Residual-style Fusion Strategy

$$y = f_1(x) + \lambda\big(f_2(x) - f_1(x)\big) \tag{S1}$$

where $\lambda \in [0,1]$ controls the contribution of the auxiliary pathway. This formulation can be equivalently expressed as a weighted combination:

$$y = (1-\lambda)f_1(x) + \lambda f_2(x). \tag{S2}$$

Although Eq. (S2) can be expressed in a form similar to a weighted combination, the role of the auxiliary pathway in the proposed architecture differs from that of a standard ensemble component. In this framework, the upper pathway serves a standard ensemble component. In this framework, the upper pathway serves as the primary reference stream, while the lower pathway is designed to capture differential information relative to this reference. Accordingly, the fusion coefficient λ governs the strength of this corrective adjustment, rather than representing a symmetric contribution from independently optimized predictors.

However, the residual-style representation emphasizes that Model 1 serves as the reference pathway, while Model 2 contributes only the differential (residual) information relative to the dominant inference. This design choice directly corresponds to the visual structure of Figure 4, in which the darker upper pathway encodes primary clinical risk patterns and the lighter lower pathway encodes secondary or corrective patterns.

The residual fusion strategy offers several advantages. First, it preserves the stability of the dominant inference flow while allowing selective correction by the auxiliary pathway. Second, it reduces excessive variance that may arise from naive ensemble averaging. Third, it enables explicit interpretation of how complementary representations modify the primary risk estimate. It should be noted that this regularization does not explicitly enforce representation-level orthogonality or independence.

5.2.4. Regularization-based Separation of Dual Pathways

To encourage differentiation between the two inference pathways during joint optimization, we introduce a pathway separation regularizer defined in parameter space. This regularization penalizes similarity between the parameter sets of the two pathways. However, it should be noted that parameter-space distance does not guarantee functional or representation-level complementarity. Instead, the role of this regularizer is to discourage trivial convergence of the two pathways toward similar parameter configurations, thereby maintaining pathway differentiation at the optimization level. In this sense, the regularizer acts as a practical inductive bias to prevent pathway collapse, rather than as a strict mechanism for enforcing complementary representations. The overall loss function is defined as follows.

$$L = L_{pred}(y, \boldsymbol{y}^*) + \beta\|\omega_1 - \omega_2\|_2^2 \tag{S3}$$

where $L_{pred}(y, y^*)$denotes the prediction loss (e.g., mean squared error or cross-entropy), $y^*$is the ground-truth label, and β is a regularization coefficient that controls the degree of separation between the two parameter sets.

This regularization term discourages the two pathways from converging to identical parameter configurations while preventing excessive divergence. As a result, Model 1 is guided to learn dominant

and globally consistent clinical risk patterns, whereas Model 2 is encouraged to capture residual, heterogeneous, or less frequent patterns that may not be sufficiently represented in the primary pathway. Through this mechanism, the two pathways form a complementary relationship rather than a competitive one.

5.2.5. Interpretation and Design Implications

By combining residual-style fusion with regularization-based separation, the proposed differential dual-drive architecture achieves a balance between robustness and expressiveness. The dominant pathway ensures stable risk estimation grounded in major clinical trends, while the auxiliary pathway refines this estimate by incorporating differential information derived from the same clinical inputs. This structural separation enhances robustness to measurement variability and improves the model's ability to capture heterogeneous interactions among biomarkers.

Importantly, this architecture aligns with the clinical interpretation of disease risk assessment, where a primary diagnosis is often established based on dominant risk factors and subsequently refined through secondary considerations. Consequently, the proposed model not only improves predictive performance but also provides a structurally interpretable framework that supports downstream expert-system-based reasoning and decision support.

5.2.6. Optimization and Training Details

The proposed differential dual-drive model is trained in an end-to-end manner using supervised learning. Let y^*denote the ground-truth disease label or risk score. The training objective consists of two components: a prediction loss and a regularization-based separation term.

The overall loss function is defined as:

$$L = L_{pred}(y, y^*) + \beta \|\omega_1 - \omega_2\|_2^2, \quad \text{(S4)}$$

where L_{pred} represents the prediction loss (e.g., mean squared error for regression-based risk estimation or cross-entropy loss for classification), and ω_1 and ω_2 denote the learnable parameters of Model 1 and Model 2 drive-ways, respectively. The regularization coefficient β controls the degree of enforced separation between the two inference pathways.

During training, gradients are jointly propagated through both pathways and the fusion module. The residual-style fusion parameter λ is treated as a fixed hyperparameter or learned scalar, depending on experimental configuration. The hyperparameters were selected empirically to ensure stable training, and the model behavior was not highly sensitive with in a reasonable range .This joint optimization encourages Model 1 to converge toward stable and dominant clinical risk representations, while Model 2 learns auxiliary patterns that refine or correct the dominant inference.

Standard optimization techniques such as stochastic gradient descent or adaptive optimizers (e.g., Adam) are employed, with early stopping and validation-based model selection to prevent overfitting. Importantly, the separation regularization ensures that performance gains are not achieved by redundant duplication of representations but by learning complementary inference behaviors across pathways.

Based on this optimization and regularization strategy, the proposed inference framework was subsequently applied to disease prediction through a structured sequential procedure.

In addition, for the prediction of a specific disease, this study follows a sequential analytical procedure as outlined below. First, an initial input vector is constructed by integrating individual anthropometric information with measurement data obtained from blood examinations. Based on this optimization and regularization strategy, the proposed inference framework is applied to disease prediction through a structured sequential procedure. For a specific disease prediction task, an initial input vector X is constructed from individual anthropometric and demographic information obtained from lifelog data, reflecting short-term physiological conditions. The corresponding blood examination measurements— LDL , HDL , TG , and TC —a are treated as supervised target biomarkers during the training phase rather than direct input features. Based on the input vector X, iterative learning processes are conducted

to generate stage-wise lipid predictions. For example, an intermediate LDL estimate, denoted as $\mathrm{LDL}_{\mathrm{PRE}}^{(1)}$, is recursively reintroduced into the learning model together with the original input X to generate subsequent-stage predictions. This sequential reuse of predicted biomarkers enables structured interdependency modeling across lipid variables within the proposed dual drive-way framework.

As illustrated in Figure 3, anthropometric information derived from lifelog data represents short-term physiological states, whereas clinically established diagnostic criteria and reference ranges correspond to long-term physiological facts. These long-term facts are not directly used as raw numerical inputs but are incorporated through rule-based inference mechanisms that guide the interpretation of predicted lipid biomarkers.

The proposed inference engine integrates short-term anthropometric inputs, sequentially estimated lipid biomarkers, and clinically defined rule-based conditions to detect abnormal health states and generate appropriate diagnostic alerts.

As shown in Figure 3, the proposed model employs more than 15 anthropometric features together with blood-based measurements (LDL, HDL, TG, and TC) as input variables and performs sequential prediction over a total of five stages. In the first stage, an initial predicted value, LDL_{PRE}, is obtained using the M_{LDL_X} model. Subsequently, this predicted value is used as an auxiliary input to iteratively train models for HDL_{PRE} and LDL_{PRE}, following a recursive learning structure. Through this staged prediction process, a final output, denoted as LDL_{PRE_LAST}, is obtained.

In summary, this study presents a disease risk prediction process that integrates individual anthropometric data and blood test results as combined inputs to estimate disease risk, including metabolic syndrome. To this end, deep learning–based artificial intelligence models are combined with semi-supervised learning techniques to perform individualized LDL prediction. This approach demonstrates a staged inference structure that enables personalized health risk assessment through the sequential integration of physiological measurements and clinical knowledge. The effect of λ and β is further analyzed in Section 6.4.

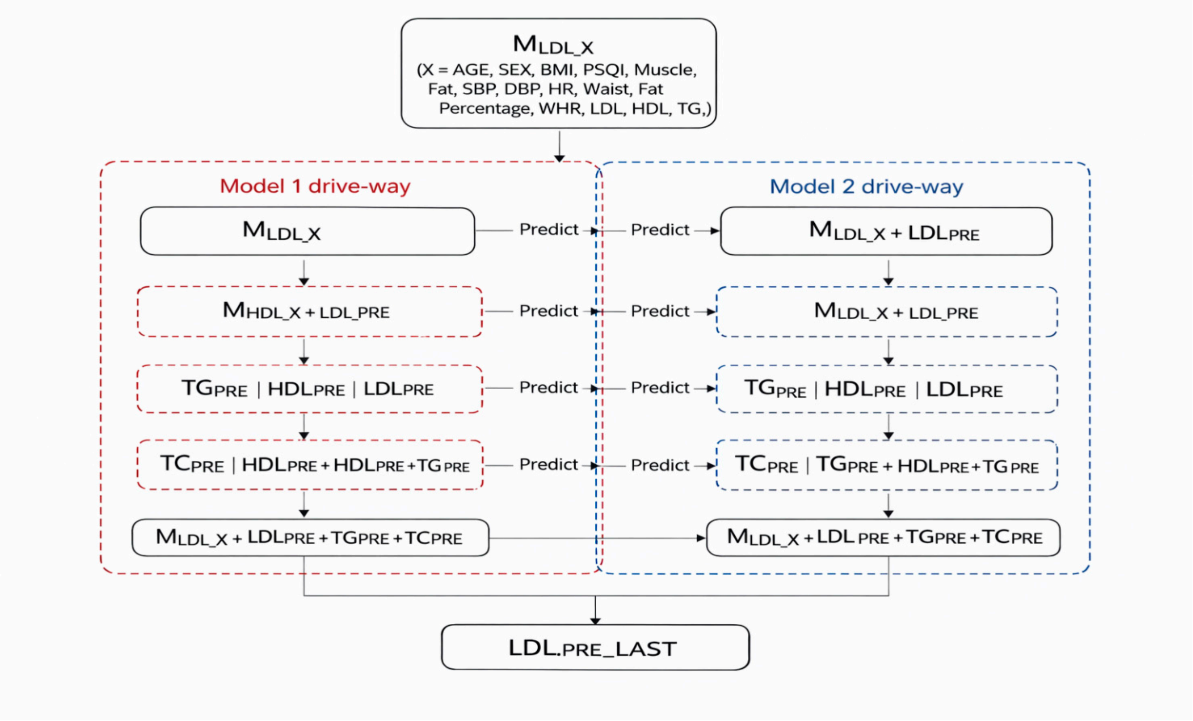

Figure-S3. Sequential and parallel drive-way architecture for LDL prediction. The model sequentially starts from M_{LDL_X} and subsequently forms two bounded parallel drive-ways (Model 1 drive-way and Model 2 drive-way) that iteratively incorporate previously predicted lipid variables, resulting in a single final output LDL_{PRE_LAST}.

This supplementary document provides an extended description of the expert system architecture introduced in **Section 6** of the main text.

6.1. Ablation Study under Simplified Experimental Setting

6.1.1 Theoretical Justification of Sequential Dual Drive-way Separation

The proposed sequential dual drive-way architecture is designed to explicitly separate rule-guided inference and data-driven learning, rather than allowing both components to be implicitly entangled within a single representation space. This separation plays a critical role in improving stability, clinical reliability, and generalization.

Let $f_r(\cdot)$ denote the rule-guided inference pathway, which encodes clinically validated constraints and long-term medical knowledge, and let $f_d(\cdot)$ denote the data-driven pathway, which learns statistical patterns from observed data. The final prediction $\hat{y}$ is obtained as

$$\hat{y} = g\big(f_r(x), f_d(x)\big), \tag{S5}$$

Equation (S5) defines the final prediction as a fusion of rule-guided and data-driven inference pathways. f(x), f(x)

Here (·) represents a fusion function. In the absence of explicit separation, both pathways tend to optimize the same empirical loss, leading to feature interference and overfitting:

$$\min_{\theta_r, \theta_d} \mathcal{L}\big(y, g(f_r(x), f_d(x))\big), \tag{S6}$$

Equation (S6) describes the joint optimization of both pathways under a shared loss function, which may lead to feature interference and overfitting when explicit separation is not enforced. In this case, clinically meaningful constraints may be overridden by spurious correlations in data.

To address this issue, the proposed model introduces separation regularization, encouraging functional disentanglement between the two pathways:

$$\mathcal{L}_{total} = \mathcal{L}_{task} + \lambda \mathcal{R}\big(f_r(x), f_d(x)\big), \tag{S7}$$

Equation (S7) introduces a regularization term that encourages functional separation between the two pathways, thereby improving stability and preserving clinically meaningful constraints.

Here, $\mathcal{R}(\cdot)$ penalizes redundant or highly correlated representations between the rule-guided and data-driven pathways.

This formulation allows $f_r(\cdot)$ to act as a stabilizing constraint that suppresses physiologically implausible predictions, while $f_d(\cdot)$ focuses on refining numerical precision. As a result, the model achieves improved high-risk sensitivity and reduced overfitting, as empirically demonstrated in Figure 5 in main material.

6.1.2. Hyperparameter Sensitivity Analysis

It should be noted that the ablation study was conducted under a simplified experimental setting (e.g., binary or reduced-class classification). Therefore, the reported accuracy values are not directly comparable to those of the primary four-class classification task.

To further validate the design of the proposed dual drive-way architecture, we analyze the sensitivity of the model to two key hyperparameters: the fusion coefficient λ and the regularization weight β.

The fusion coefficient λ controls the contribution of the auxiliary pathway in the residual-style fusion. As described in Section 5.2, the auxiliary pathway is designed to provide corrective (residual-like) information relative to the dominant pathway. To evaluate its effect, λ was varied over a predefined range, and the corresponding classification performance was measured. These results are obtained under the simplified experimental setting described above.

The results indicate that the model achieves stable performance within a moderate range of λ values. When λ is too small, the auxiliary pathway contributes minimally, and the model behaves similarly to a single dominant pathway. Conversely, when λ becomes large, the influence of the auxiliary pathway increases, which may introduce instability due to excessive correction. In our experiments, $\lambda = 0.3$ provided the best balance between stability and corrective contribution, and was therefore selected for all reported results.

The regularization weight β controls the degree of separation between the two pathways during joint optimization. To examine its impact, β was varied across multiple orders of magnitude. The results show that small values of β are sufficient to prevent trivial convergence of the two pathways toward similar parameter configurations. In contrast, larger values of β impose excessive constraints on the parameter space, leading to degraded performance.

Based on this analysis, $\beta = 0.001$ was selected as it provided stable performance while avoiding over-regularization. It should be noted that this regularization term does not guarantee representation-level complementarity, but serves as an optimization-level constraint to maintain pathway differentiation during training.

Overall, the ablation results demonstrate that the proposed framework is not highly sensitive to precise hyperparameter tuning within a reasonable range under the simplified experimental setting, indicating robustness of the dual drive-way architecture.

Table-S1. Ablation results under a simplified experimental setting, showing the effects of the fusion coefficient (λ) and the regularization weight (β) on classification performance. Accuracy and F1-scores are reported for a reduced evaluation task and are not directly comparable to the primary four-class classification results.

(a) Effect of λ on model performance

λ value	Accuracy (%)	F1-score
0.1	81.2	0.812
0.3	84.5	0.845 (Best)
0.5	83.7	0.837
0.7	81.9	0.819

(b) Effect of β on model performance

β value	Accuracy (%)	F1-score
0.0001	83.8	0.838
0.001	84.5	0.845 (Best)
0.01	83.1	0.831
0.1	80.6	0.806

Table-S1 presents the ablation results for the fusion coefficient (λ) and the regularization weight (β). The results indicate that moderate values of λ provide a balance between the dominant and auxiliary pathways, while small values of β are sufficient to prevent pathway collapse without introducing excessive constraints.

These results are obtained under the simplified experimental setting and therefore reflect relative performance trends rather than absolute performance comparable to the primary four-class classification task. Thus, the ablation study is intended to validate architectural design choices rather

than to directly compare predictive performance with the main evaluation results. These findings indicate that the proposed dual drive-way architecture is robust to hyperparameter variations within a reasonable range.
